# Oral administration of Manuka honey induces IFNγ-dependent resistance to tumor growth that correlates with beneficial modulation of gut microbiota composition

**DOI:** 10.3389/fimmu.2024.1354297

**Published:** 2024-02-20

**Authors:** Razan J. Masad, Ienas Idriss, Yassir A. Mohamed, Ashraf Al-Sbiei, Ghada Bashir, Farah Al-Marzooq, Abeer Altahrawi, Maria J. Fernandez-Cabezudo, Basel K. Al-Ramadi

**Affiliations:** ^1^ Department of Medical Microbiology and Immunology, College of Medicine and Health Sciences, United Arab Emirates University, Al Ain, United Arab Emirates; ^2^ Department of Biochemistry and Molecular Biology, College of Medicine and Health Sciences, United Arab Emirates University, Al Ain, United Arab Emirates; ^3^ Department of Pathology, College of Medicine and Health Sciences, United Arab Emirates University, Al Ain, United Arab Emirates; ^4^ Zayed Center for Health Sciences, United Arab Emirates University, Al Ain, United Arab Emirates; ^5^ ASPIRE Precision Medicine Research Institute Abu Dhabi, United Arab Emirates University, Al Ain, United Arab Emirates

**Keywords:** Manuka honey, immunomodulation, type I/II IFN, tumor immunogenicity, colorectal cancer

## Abstract

**Background:**

To investigate the potential of Manuka honey (MH) as an immunomodulatory agent in colorectal cancer (CRC) and dissect the underlying molecular and cellular mechanisms.

**Methods:**

MH was administered orally over a 4 week-period. The effect of MH treatment on microbiota composition was studied using 16S rRNA sequencing of fecal pellets collected before and after treatment. Pretreated mice were implanted with CRC cells and followed for tumor growth. Tumors and lymphoid organs were analyzed by flow cytometry (FACS), immunohistochemistry and qRT-PCR. Efficacy of MH was also assessed in a therapeutic setting, with oral treatment initiated after tumor implantation. We utilized IFNγ-deficient mice to determine the importance of interferon signaling in MH-induced immunomodulation.

**Results:**

Pretreatment with MH enhanced anti-tumor responses leading to suppression of tumor growth. Evidence for enhanced tumor immunogenicity included upregulated MHC class-II on intratumoral macrophages, enhanced MHC class-I expression on tumor cells and increased infiltration of effector T cells into the tumor microenvironment. Importantly, oral MH was also effective in retarding tumor growth when given therapeutically. Transcriptomic analysis of tumor tissue highlighted changes in the expression of various chemokines and inflammatory cytokines that drive the observed changes in tumor immunogenicity. The immunomodulatory capacity of MH was abrogated in IFNγ-deficient mice. Finally, bacterial 16S rRNA sequencing demonstrated that oral MH treatment induced unique changes in gut microbiota that may well underlie the IFN-dependent enhancement in tumor immunogenicity.

**Conclusion:**

Our findings highlight the immunostimulatory properties of MH and demonstrate its potential utilization in cancer prevention and treatment.

## Introduction

Cancer represents a crucial global health concern, accounting for 10 million deaths annually (1). Cancer growth results from a multistep process during which cells acquire multiple mutations, eventually leading to continuous cellular growth and division. Although several factors can contribute to cancer development, a compromised immune system is widely recognized as a dominant contributor to the onset and progression of cancer (2, 3).

The role of the immune system in cancer is illustrated by its ability to eradicate emerging transformed cells once they arise, a concept known as “cancer immunosurveillance” (4). However, tumor cells are capable of acquiring characteristics and strategies by which they can evade the immune system and consequently progress in their growth (5). In light of the vital role of the immune system in cancer development and progression, there is a rising interest in employing cancer immune preventive agents to amplify immune responses and reduce cancer susceptibility in healthy individuals.

There is a growing body of evidence suggesting that different types of honey have anti-cancer properties (6). Previously, our lab and others demonstrated the potential of Manuka honey (MH) to impede the growth of various types of human and murine cancer cell lines (7–9) and revealed the underlying molecular mechanisms of its anti-tumor action (8, 10). MH has also been described to possess immunomodulatory properties (11). While some studies highlighted the potential of MH as an anti-inflammatory agent (12, 13), others demonstrated that MH also exhibits pro-inflammatory properties (14–19).

In our previous work, we demonstrated that MH can trigger the activation of macrophages by inducing the expression of pro-inflammatory cytokines, such as TNF-α and IL-1β, and the chemokines CXCL2 and CCL2 which are potent chemoattractants of myeloid cells (15). Additionally, when administered intraperitoneally, MH elicited a peritoneal immune response characterized by a significant increase in the recruitment of neutrophils and an enhancement in the functional maturation of peritoneal macrophages (15). In the present study, we investigated the effect of oral administration of MH, given as part of a preventive or therapeutic regimen, on the host immune system and its potential to modulate anti-tumor immune responses in implantable murine colorectal cancer (CRC) models. Several reports demonstrated that alterations in the composition of gut microbiota and their translocation to secondary lymphoid organs can stimulate immune responses against tumors by influencing various cell types such as CD8^+^ and CD4^+^ T cells as well as tumor-associated myeloid cells (20, 21). Therefore, we also assessed the potential changes in microbiota composition following MH treatment in this study. Our findings provide compelling evidence that supports a role for MH as an immunomodulatory anti-tumor agent, highlighting its potential use in cancer prevention and treatment.

## Materials and methods

### Cell line and reagents

The murine CT26 colon carcinoma cell line was a kind gift from Dr. Siegfried Weiss (Helmholtz Centre for Infection Research, Braunschweig, Germany). The colon adenocarcinoma MC38 cell line was provided by Prof. Jo Van Ginderachter (Vrije University Brussel, Belgium). Cells were maintained as previously described (7, 22). Manuka honey (UMF^®^ 20+ from ApiHealth, Auckland, New Zealand) was used in the current study and diluted in distilled water under aseptic conditions. MH is composed mainly (~76%) of a mixture of sugars (fructose, glucose, maltose, sucrose and galactose) together with a significant component of bioactive compounds, including phenolics and flavonoids (6). As a control for MH, a sugar solution, designated sugar control (SC), containing equivalent concentrations of the three major sugars in honey (38.2% fructose, 31.3% glucose, and 1.3% sucrose) was used (Sigma, St. Louis, MO, USA) (8).

### Experimental animals

BALB/c and C57BL/6 mice were purchased from the Jackson Laboratory (Bar Harbor, ME, USA). IFNγ-deficient (IFNγ^-/-^) mice were purchased from the Jackson laboratories (USA) and have been described (23). All animals were bred in the animal facility of the College of Medicine and Health Sciences, United Arab Emirates University. For the current study, male mice at the age of 8-10 weeks were used. Female mice were not used to avoid potential physiological variability associated with the estrous cycle. Mice received rodent chow and water ad libitum and were maintained 5-6 mice per cage in a standard 12 h light/12 h dark cycle at a temperature of 21°C with 40–57% humidity. All studies involving animals were carried out in accordance with, and after approval of the Animal Research Ethics Committee of the United Arab Emirates University (Protocols #A12-13 and ERA-2019-5853).

### Oral treatment and tumor studies

BALB/c mice of comparable age and weight were randomly divided into two groups. Mice were gavaged daily with 0.2 mL of a water solution containing 70% SC or 70% MH (w/v). After 4 weeks of treatment, mice were euthanized, and their mesenteric lymph nodes (MLNs), inguinal lymph nodes (ILNs), and spleens were excised for further analysis.

For the tumor model studies, BALB/c or C57BL/6 mice were treated with SC or MH for 4 weeks, then subcutaneously inoculated with CT26 (2×10^5^/mouse) or MC38 (1×10^5^/mouse) cells, respectively, in the right flank. Tumor dimensions (width and length) were measured using a digital caliper twice a week, and tumor volume was calculated using the formula: tumor volume = (L×W^2^)/2, as detailed elsewhere (24, 25). Mice were euthanized 21 days post-implantation, and their tumors were excised for further analysis. In other experiments, oral MH was administered as a therapeutic regimen by first implanting tumor cells and then starting daily MH administration from day 3 post implantation for a total period of up to 3 or 4 weeks, as indicated. For these studies, we utilized wild-type C57BL/6 and IFNγ^-/-^ mice.

### Processing of lymphoid organs and tumors

Single cell suspensions were prepared from the spleens, MLNs, and ILNs by mechanical dissociation as previously described (26). Tumors were processed using a previously described method, with modification (27). Briefly, dissected tumor tissues were subjected to mechanical and enzymatic digestion in gentleMACS C-tubes (Miltenyi Biotec, Germany) using a tumor dissociation kit (Miltenyi Biotec) and the GentleMACS dissociator (Miltenyi Biotec), according to manufacturer’s instructions. Tumor-infiltrating leukocytes (TILs) were subsequently purified from tumor cell suspensions using magnetic CD45^+^ microbeads and the autoMACS cell separator, according to the manufacturer’s protocol (Miltenyi Biotec).

### Flow cytometric analysis

Analysis of MLN, ILN, spleen, and tumor cells was carried out using multi-color flow cytometry, following our standard protocol (22, 27). The following antibodies (all purchased from Biolegend, San Diego, CA, USA) were used in the current study: anti-CD45-APC (Cat# 103112), anti-CD19-PE (Cat# 115508), anti-CD19-PE-Texas Red (Cat# 115554), anti-CD3-BV785 (Cat# 100232), anti-CD4-FITC (Cat# 100509), anti-CD8-APC-Cy7 (Cat# 100714), anti-CD8-APC (Cat# 100712), anti-CD11b-Alexa Flour-488 (Cat# 101217), anti- CD11c-PE (Cat# 117308), anti- Ly6G-BV605 (Cat# 127639), Ly-6A/E (Sca-1)-PE-Texas Red (Cat# 108138), anti-MHC II (I-A/I-E)-BV785 (Cat# 107645), anti-MHC I H-2K^d^ -BV421 (Cat# 116623). Non-viable cells from tumors were excluded using 7-AAD viability dye (Biolegend) and non-viable cells from spleens, MLNs, and ILNs were excluded using Zombie Aqua dye (Biolegend). Data were collected on 10,000-50,000 cells (depending on the organ) using a FACSCelesta flow cytometer (BD Biosciences, Mountain View, CA, USA) and analyzed using FlowJo software (BD Biosciences).

### Immunohistochemical analysis

Immunohistochemical staining was performed on tumor tissue sections as per established protocols in our laboratory (22, 28). Sections were incubated overnight with specific monoclonal antibodies to CD8 (ab209775; Abcam, UK), CD4 (ab183685; Abcam), or granzyme-B (44153S; Cell Signaling Technology, Danvers, MA, USA), after which they were incubated with HRP-conjugated goat polyclonal secondary antibody for 45 min at room temperature. Sections were then developed using DAB chromogen substrate (Dako, Carpinteria, CA, USA), counterstained with hematoxylin, and examined using an Olympus BX51 microscope (Olympus Corporation, Japan) at 40× magnification. The positive cells were counted in 10-20 randomly selected high-power fields (HPF), and the average count was calculated.

### Quantitative real-time PCR

qRT-PCR was carried out essentially as previously detailed (22, 27). We used premade TaqMan primers and probes (Applied Biosystems, Foster City, CA, USA) for the following genes, CXCL1 (Mm04207460_m1), CXCL2 (Mm00436450_m1), CXCL10 (Mm99999072_m1), IFN-γ (Mm01168134_m1), and granzyme B (Mm00442834_m1). mRNA levels of target genes were normalized according to the comparative ΔCq method to respective mRNA levels of the housekeeping gene HPRT (Mm01545399_m1). The expression of the target gene is reported as the level of expression relative to HPRT and presented as fold change relative to control mice.

### Fecal sample collection and DNA extraction

DNA was extracted from stool samples using the QIAamp Fast DNA Stool Mini Kit (Qiagen, Valencia, CA, USA), following standard protocol. DNA concentration was measured using the NanoDrop 1000 Spectrophotometer (ThermoFisher Scientific, Waltham, MA, USA).

### Bacterial 16S rRNA gene amplicon sequencing

16S Metagenomic Sequencing kit (Illumina, San Diego, CA, USA) was used for library preparation. V3–V4 hypervariable regions of the bacterial 16S rRNA gene were amplified using the primers (5′-CCTACGGGNGGCWGCAG-3′ and 5′ GACTACHVGGGTATCTAATCC-3′) provided by the manufacturer and following the recommended protocol as described before (29). Library concentration was assessed by Qubit Fluorometric Quantitation (Invitrogen, Waltham, MA, USA). Short-read paired-end amplicon sequencing was performed using Illumina^®^ MiSeq Instrument for 600 cycles.

### Bioinformatic analysis

Processing of sequencing reads (adaptor trimming and filtering of low-quality reads) followed by taxonomic classification were done using Quantitative Insights Into Microbial Ecology version 2 (QIIME2) software suite (30). After the identification of Operational Taxonomic Units (OTUs), downstream analyses were carried out in RStudio (v 4.1.2). Diversity was measured using BiodiversityR (v 2.15-2) and plotted by ggplot2 (v. 4.1.3). Alpha diversity measures (Observed OUT, CHAO1, Shannon’s Diversity, and Simpson’s Diversity indices) were compared between the groups using Mann-Whitney U test. For beta diversity, principal coordinate analysis based on Jaccard and Bray Curtis dissimilarity metrics was used to assess differences between the groups using non-parametric multivariate analysis of variance (PERMANOVA). Linear discriminant analysis (LDA) effect size (LEfSe) was used to detect biomarkers from microbial profiles (31) using the Microbiome Analyst 2.0 platform (McGill, Canada), which was also used to generate the graph of relative abundance and the heatmap for groups comparison (32). Venn diagrams were generated to compare the taxa exhibiting significant differences based on the LDA analysis for the identification of shared and unique OTUs (33).

### Statistical analysis

All statistical analyses were performed using GraphPad Prism 9.0 (San Diego, CA, USA). Statistical significance between control and treated groups was determined using 2-way ANOVA or the unpaired, two-tailed Student’s t-test, as indicated. In all analyses, *p* < 0.05 was considered statistically significant * (*p* < 0.05), ** (*p* < 0.01), *** (*p* < 0.001) and **** (*p* < 0.0001).

## Results

### Oral administration of MH induces functional alterations in host immune responses

We have previously demonstrated the ability of i.p. administration of MH to effect changes in the immune system via inducing the recruitment of neutrophils into the peritoneal cavity and the maturation of peritoneal macrophages (15). In our efforts to apply a more physiological route of administration that would be safe and more applicable to humans, we investigated the effect of repeated oral administrations of MH on the immune system of BALB/c mice. Based on our previous experience, a solution of 50-70% MH (w/v) is suitable for *in vivo* use in mice (7). Naïve BALB/c mice were orally gavaged with water (control group), sugar control (SC) solution, or MH for 4 weeks. To address if repeated oral doses of MH are associated with any adverse events, body weights were determined in treated animals over the 4-week period. Baseline body weights were recorded before starting the treatment (Means ± SEM = 22.97 ± 0.397, 23.63 ± 0.783, 23.54 ± 0.818 g for the water, SC and MH groups, respectively) and at weekly intervals after treatment. The percentage change in body weight from baseline was then calculated. The results indicated that, in comparison with water-treated and SC-treated groups, treatment with MH over 4 weeks did not affect normal weight gain, with all 3 experimental groups showing comparable levels of body weight gain over the treatment period (**Supplementary Figure 1A**). Accordingly, we selected SC as the control for all subsequent experiments. The potential effect of oral MH on blood glucose levels was also investigated by determining non-fasting glucose levels in blood samples of randomly-selected mice collected on a weekly basis in SC or MH-treated groups. The average random glucose blood level in untreated, age-matched, control mice is 172.0 ± 14.9 mg/dL (mean ± SD). At the end of the treatment period, we observed that MH administration did not alter the blood glucose levels, with both SC and MH groups showing comparable glucose levels that lie within the normal range (<200 mg/dL) (**Supplementary Figure 1B**). Thus, no apparent negative effects were evident following oral administration of MH.

The capacity of MH to effect changes in the immune system was next investigated. Different peripheral lymphoid tissues including gut-draining MLNs, ILNs, and spleens were collected, and their weights and absolute cell counts were recorded. Our results indicated that MH administration did not alter the weights or the total cell counts of the collected tissues in comparison to SC-treated mice (**Supplementary Figures 1C–H**).

Next, multi-color flow cytometry was utilized to analyze the cellular changes in the collected tissues following MH administration. The gating strategy employed to identify the major immune subpopulations is shown in (**Supplementary Figure 2**). FACS data indicated that MH administration did not lead to alterations in the cellular landscape of MLNs, ILNs, or spleens between SC or MH-treated mice (**Supplementary Figure 3**).

**Figure 1:**
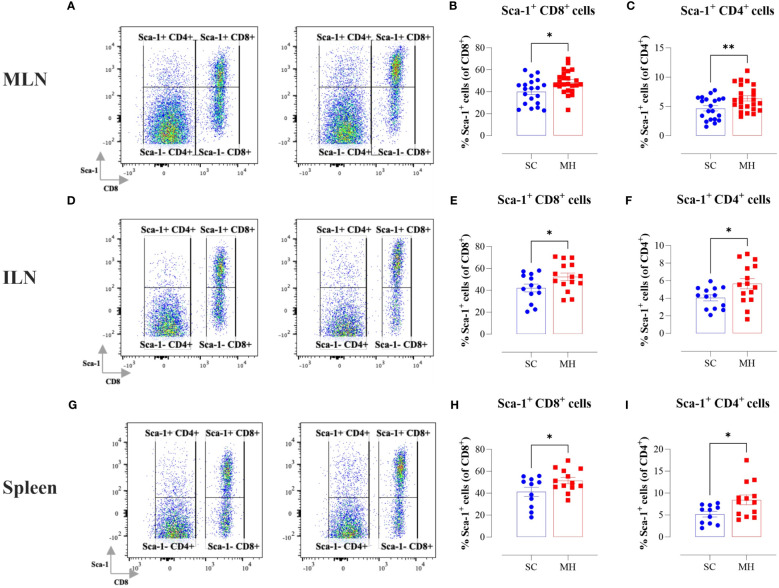
Lymphocyte activation in lymphoid tissues following MH treatment. BALB/c mice were orally gavaged with either SC or MH for 4 weeks. Following treatment, lymphoid organs were processed for flow cytometry analysis. Cells from MLNs **(A–C)**, ILNs **(D–F)**, and spleens **(G–I)** were analyzed to quantify the percentage of Sca-1^+^ CD8^+^ T cells **(B, E, H)**, and Sca-1^+^ CD4^+^ T cells **(C, F, I)**. Representative dot plots are shown in **(A, D, G)**. The values for individual mice and mean ± SEM are shown. The data is pooled from 5 **(A–C)**, 4 **(D–F)**, and 3 independent experiments **(G–I)**. *p* values were calculated using the unpaired Student’s t-test, * (*p* ≤ 0.05), and ** (*p* ≤ 0.01).

In the context of our previous findings demonstrating a functional maturation of macrophages that was observed following i.p. administration of MH, we next sought to investigate if similar alterations were induced following oral MH administration. Upregulation of MHC class II proteins on myeloid cells is a key event that is induced in response to activation through type I/II interferon signaling pathways (34, 35). Therefore, we investigated whether oral administration of MH can induce any alterations within the cellular landscape of the peripheral lymphoid tissues, including MLN, ILN, and spleen. Given that the majority of cells in these tissues comprise T and B lymphocytes, we focused on analyzing changes in the expression of proteins known to be induced by type I/II IFNs. One of the well-known IFN-inducible genes is *Ly6a*, which encodes for Sca-1 protein on T lymphocytes (36–38). The results of the flow cytometric analysis showed that oral administration of MH led to a significant increase in Sca-1 expression on both CD8^+^ and CD4^+^ T cells (**Figure 1**). The percentage of Sca-1-positive CD8^+^ T cells in MLNs, ILNs, and spleens increased by 21.6%, 24.4%, and 24.7%, respectively in MH-treated mice in comparison to SC-treated mice (**Figures 1A, B, D, E, G, H**). Similarly, the percentage of Sca-1-positive CD4^+^ T cells increased by 36.5%, 39.8%, and 62.9% in the same three organs, respectively (**Figures 1A, C, D, F, G, I**). These results show that oral MH administration induced IFN-dependent responses in T cells, both at the level of the gastrointestinal tract as well as in systemic lymphoid organs.

### Oral administration of MH retards the growth of implanted tumors

**Figure 2:**
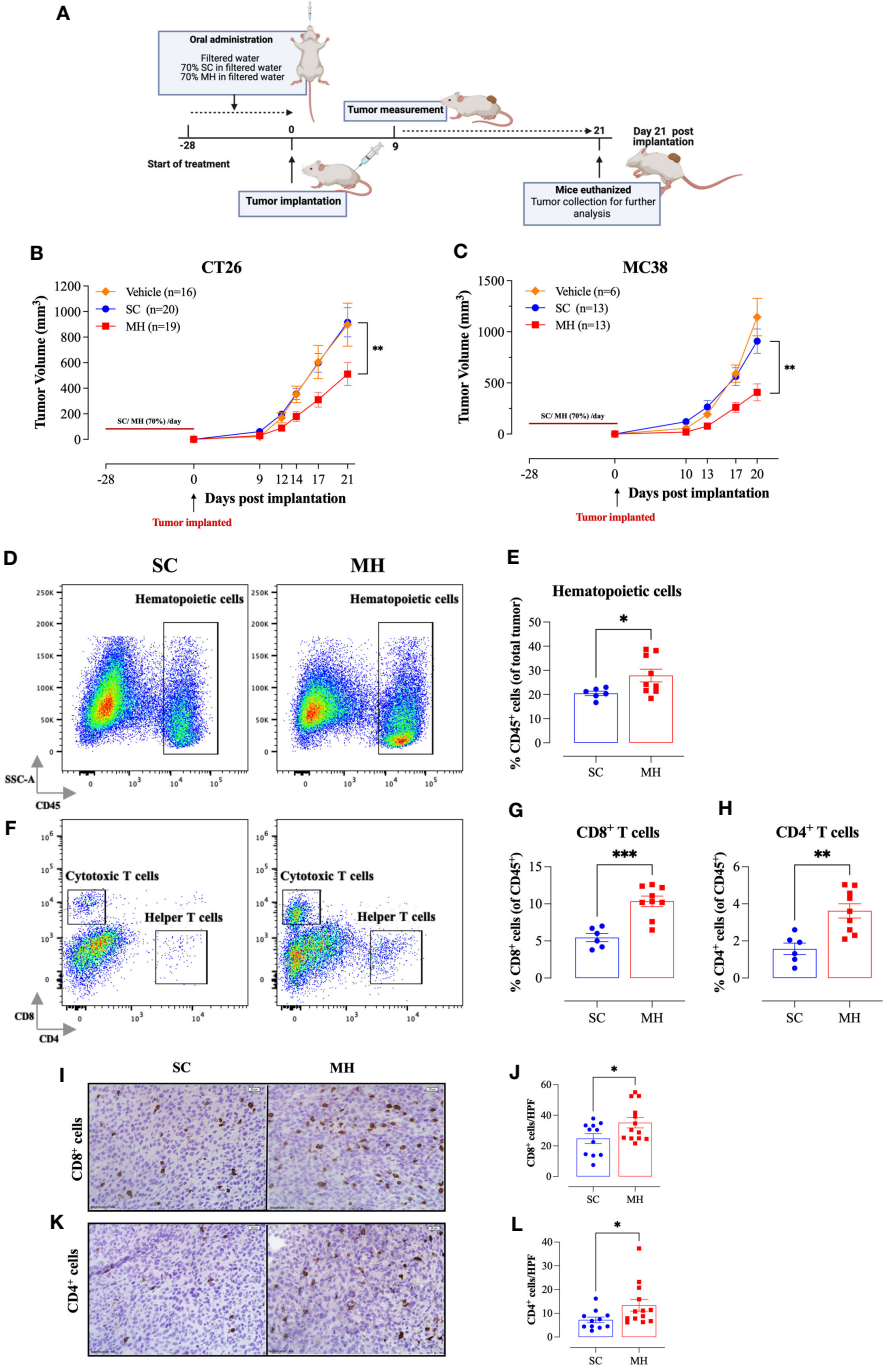
Retardation of tumor growth in MH pre-treated mice correlates with enhanced tumor infiltration by immune cells. **(A)** A schematic diagram describing the preventative model treatment protocol. Mice were orally gavaged daily with filtered water, 70% SC or 70% MH for 4 consecutive weeks. Following the treatment period, CT26 or MC38 CRC cells were implanted and tumor growth was followed for the subsequent 3 weeks. Mice were euthanized on day 21 post-implantation, and tumors were excised and processed for further analysis. Tumor growth curves of CT26 **(B)** or MC38 tumor **(C)** in water-treated, SC-treated, and MH-treated mice are shown. Each data point represents the mean ± SEM of 16-20 mice, pooled from 3 individual experiments. Asterisks denote statistically significant differences between the SC-treated and MH-treated groups. *p* values were calculated using 2-way ANOVA. Resected tumors were analyzed for the extent of intratumoral immune cells by flow cytometry **(D–H)** and immunohistochemistry **(I–L)**. Representative dot plots and quantification of percentage of CD45^+^ immune cells **(D, E)**, CD8^+^ cytotoxic T cells **(F, G)**, and CD4^+^ helper T cells **(F, H)**. The values for individual mice and mean ± SEM are shown (SC: n=6, MH: n=9), pooled from 2 independent experiments. **(I–L)** Tissue sections were analyzed by immunohistochemistry to quantify the number of CD8^+^ and CD4^+^ cells. Representative images at 40× magnification (scale bar 20 μm), and the quantitative estimation of the number of CD8^+^ cells **(I, J)** and CD4^+^ cells **(K, L)** per HPF (high-power field) are presented for each group. The values for individual mice and mean ± SEM are shown (SC: n=11, MH: n=13), pooled from 3 independent experiments. Asterisks denote statistically significant differences between the MH-treated and SC-treated groups. *p* values were calculated using the unpaired Student’s t-test, * (*p* ≤ 0.05), ** (*p* ≤ 0.01), and *** (*p* ≤ 0.001).

The demonstration of the capacity of oral MH administration to activate T lymphocytes supports its potential role as an immunomodulatory agent. We reasoned that oral MH could potentially be used to boost immune responses preventatively in different disease settings. To test this hypothesis, we investigated the capacity of MH to modulate anti-tumor immune responses using two different syngeneic murine CRC models, namely CT26 tumors in BALB/c mice and MC38 tumors in C57BL/6 mice. The treatment protocol followed in this study is illustrated in **Figure 2A**. Our findings revealed that pre-treating mice with MH resulted in a significant growth retardation of both CT26 (**Figure 2B**) and MC38 (**Figure 2C**) tumors. In the CT26 model, tumors grew continuously and rapidly in mice given vehicle (H_2_O) or SC solution, reaching a mean volume ± SEM of 897 ± 169 mm^3^ and 916 ± 114 mm^3^, respectively on day 21 post-implantation (**Figure 2B**). On the other hand, mice treated with MH exhibited a significant reduction in tumor volume, with a mean of 511 ± 90 mm^3^ on day 21 post-implantation. The suppression in tumor growth was observed as early as 9 days post-implantation, and by day 21, it reached 44% compared to SC-treated mice (*p* = 0.006) (**Figure 2B**). Tumor growth in individual mice of each of the three experimental groups is shown in **Supplementary Figures 4A, C, E**. Very similar findings were observed using the MC38 tumor model (**Figure 2C**). Pre-treatment with MH resulted in 55% suppression (*p* = 0.002) in the growth of MC38 tumors by day 20 post implantation (**Figure 2C**). These results highlight a potential immune-boosting anti-tumor role for MH when given preventively.

### MH treatment induces alterations within the tumor microenvironment

To investigate the underlying mechanism for the observed MH-mediated retardation of tumor growth, we analyzed the tumor microenvironment (TME) for alterations in the cellular landscape by flow cytometry. Tumors were excised at the end of the observation period, subjected to mechanical and enzymatic digestion, and processed to a single cell suspension. The cells were then stained with different panels of mAbs to quantify the cellular constituents within the tumor tissue. The gating strategies employed to identify the cellular subpopulations are illustrated in (**Supplementary Figure 5**).

Tumor-infiltrating immune cells were identified by being positive for the pan-hematopoietic CD45 cell surface marker. There was a significant 36% increase in CD45^+^ immune cells in the tumors of MH-treated mice compared to the control group (28% *vs.* 20%, respectively) (**Figures 2D, E**). FACS analysis revealed alterations in both the phenotypic and functional characteristics of the lymphoid and myeloid subpopulations in the TME. Regarding the CD3^+^ T cell population, we observed a ~2-fold increase in the percentages of both CD8^+^ and CD4^+^ T cells (**Figures 2F–H**) following MH treatment. The increase in the infiltration of T cells was also demonstrated morphologically by immunohistochemistry, where the number of both CD8^+^ and CD4^+^ T cells was substantially increased in tumor tissue sections of MH-treated mice (**Figures 2I–L**).

**Figure 3:**
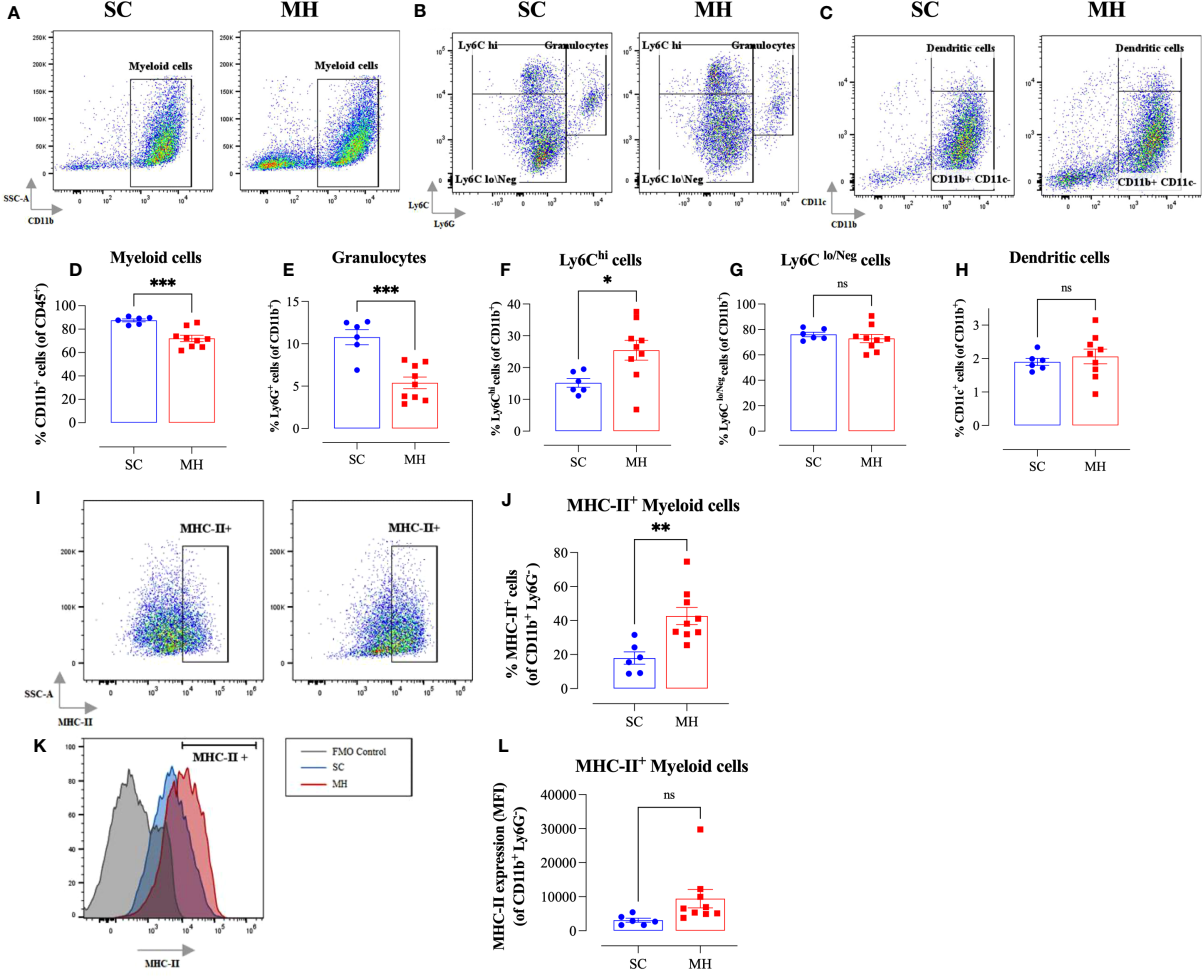
MH treatment alters intratumoral myeloid subpopulations and enhances expression of MHC class II on macrophages. BALB/c mice were orally gavaged with either 70% SC or 70% MH for 4 consecutive weeks, then implanted with CT26 tumor cells. Mice were euthanized on day 21 post-implantation, their tumors were resected, and the percentages of intratumoral myeloid cells were determined by flow cytometry. Representative dot plots and the quantification of the percentages of CD11b^+^ myeloid cells **(A, D)**, Ly6G^+^ granulocytes **(B, E)**, Ly6C^hi^ cells **(B, F)**, Ly6C^lo/Neg^ cells **(B, G)**, and CD11c^+^ dendritic cells **(C, H)**. The values for individual mice and mean ± SEM are shown (SC: n=6, MH: n=9), pooled from 2 independent experiments. Analysis of MHC class II protein expression **(I–L)**. Representative flow plots **(I)** and the quantification **(J)** of the percentage of MHC-II^+^ cells (gated on CD11b^+^ Ly6G^-^ cells) in SC-treated and MH-treated mice. **(K)** Representative overlay histograms showing MHC-II expression on CD11b^+^ Ly6G^-^ myeloid cells of SC-treated and MH-treated mice. The grey histogram indicates staining with FMO control. **(L)** Median fluorescence intensity of MHC-II^+^ CD11b^+^ Ly6G^-^ in SC-treated and MH-treated groups. The values for individual mice and mean ± SEM are shown (SC: n=6, MH: n=9), pooled from 2 independent experiments. Asterisks denote statistically significant differences between the MH-treated and SC-treated groups. *p* values were calculated using the unpaired Student’s t-test, * (*p* ≤ 0.05), ** (*p* ≤ 0.01), *** (*p* ≤ 0.001), and ns (no statistical significance, *p* > 0.05).

Further analysis using myeloid cell-specific antibodies showed that the majority (70-80%) of the gated CD45^+^ population in the TME were CD11b^+^ myeloid cells (**Figure 3A**). Interestingly, there was a significant decrease (~18%) in the percentage of intratumoral myeloid cells in the MH-treated group compared to the SC-treated group (**Figures 3A, D**). This was largely accounted for by a 50% reduction in the percentage of Ly6G^+^ granulocytes (**Figures 3B, E**), most likely representing myeloid-derived suppressor cells (39). In contrast, the percentage of Ly6C^hi^ cells increased significantly (~1.7-fold) in MH-treated mice (**Figures 3B, F**). These cells have been described to be pro-inflammatory in function and are recruited to tumor tissue in response to CCL2/CCR2 signaling (40). They further differentiate into MHC class II (MHC-II) positive or negative tumor-associated macrophages (TAM) dependent on macrophage colony-stimulating factor-mediated signals (41). In terms of the other myeloid subpopulations, there was no major change in the percentages of Ly6C^lo/Neg^ cells (**Figures 3B, G**) and dendritic cells (CD11c^+^ cells) (**Figures 3C, H**) following MH treatment.

To gain insight into the functionality of intratumoral myeloid cells, we analyzed the level of expression of MHC-II proteins on CD11b^+^ Ly6G^-^ subpopulation. There was a significant increase in the percentage of myeloid cells expressing MHC-II proteins (2.4-fold) in MH-pretreated mice (**Figures 3I, J**). Furthermore, the overall level of expression of MHC-II on myeloid cells in MH-treated group tended to be slightly elevated compared to SC-treated mice, but this difference did not reach statistical significance (*p* = 0.083) (**Figures 3I, K, L**). These data are suggestive of an enhancement in the antigen-presentation capacity of myeloid cells within the TME of MH-treated mice.

**Figure 4:**
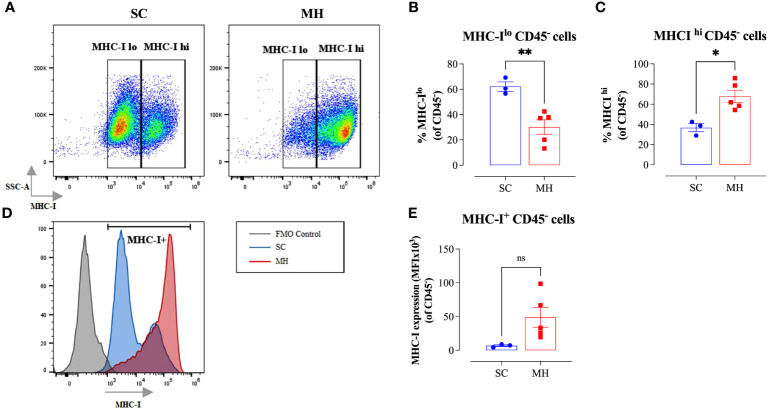
Enhancement in tumor immunogenicity as a consequence of MH administration. **(A–C)** Representative flow plots **(A)** and quantification of the percentage of MHC-I^lo^ **(B)** and MHC-I^hi^ **(C)** (gated on CD45^-^ cells) in SC-treated and MH-treated mice. **(D)** Representative overlay histograms showing MHC-I expression on CD45^-^ cells of SC-treated and MH-treated mice. The grey histogram indicates staining with FMO control **(E)** Median fluorescence intensity of MHC-I^+^ CD45^-^ cells in SC-treated and MH-treated groups. The values for individual mice and mean ± SEM are shown (SC: n=3, MH: n=5), obtained from 1 experiment. Asterisks denote statistically significant differences between MH-treated and SC-treated groups. *p* values were calculated using the unpaired Student’s t-test, * (*p* ≤ 0.05), ** (*p* ≤ 0.01), and ns (no statistical significance, *p* > 0.05).

Given the evidence of the involvement of type I/II IFN pathways in the observed functional changes in cellular function, we next analyzed whether similar alterations could be observed on the tumor cells. It is well known that the expression of MHC class I (MHC-I) proteins is regulated by type I/II IFN signaling pathways (42). Therefore, we analyzed the expression of MHC-I proteins on CD45^-^ tumor cells grown in mice after pretreatment with MH in comparison with tumor cells grown in control mice given SC solution. The results of this analysis showed that tumor cells grown in control mice showed bimodal levels of MHC-I expression, with 2 clearly discernible subpopulations being observed. The majority of these tumor cells (~62%) expressed low levels of MHC-I proteins, while the remaining population (~38%) showed high levels of MHC-I (**Figures 4A–C**). In sharp contrast, approximately 70% of tumor cells grown in mice pre-treated with MH exhibited high levels of MHC-I proteins (**Figures 4A, C**). Furthermore, a 7-fold increase in the MFI level of MHC-I proteins on tumor cells was observed following MH administration (**Figures 4D, E**). These results suggest that MH treatment indirectly enhanced the immunogenicity of tumor cells, rendering them more susceptible to killing by anti-tumor CD8^+^ T effector cells. Taken together, our findings indicate that the ability of MH to effect changes in tumor growth is linked to a series of immunomodulatory alterations within the TME.

### Altered expression of cytotoxic effector molecules and immunoregulatory mediators following MH treatment

**Figure 5:**
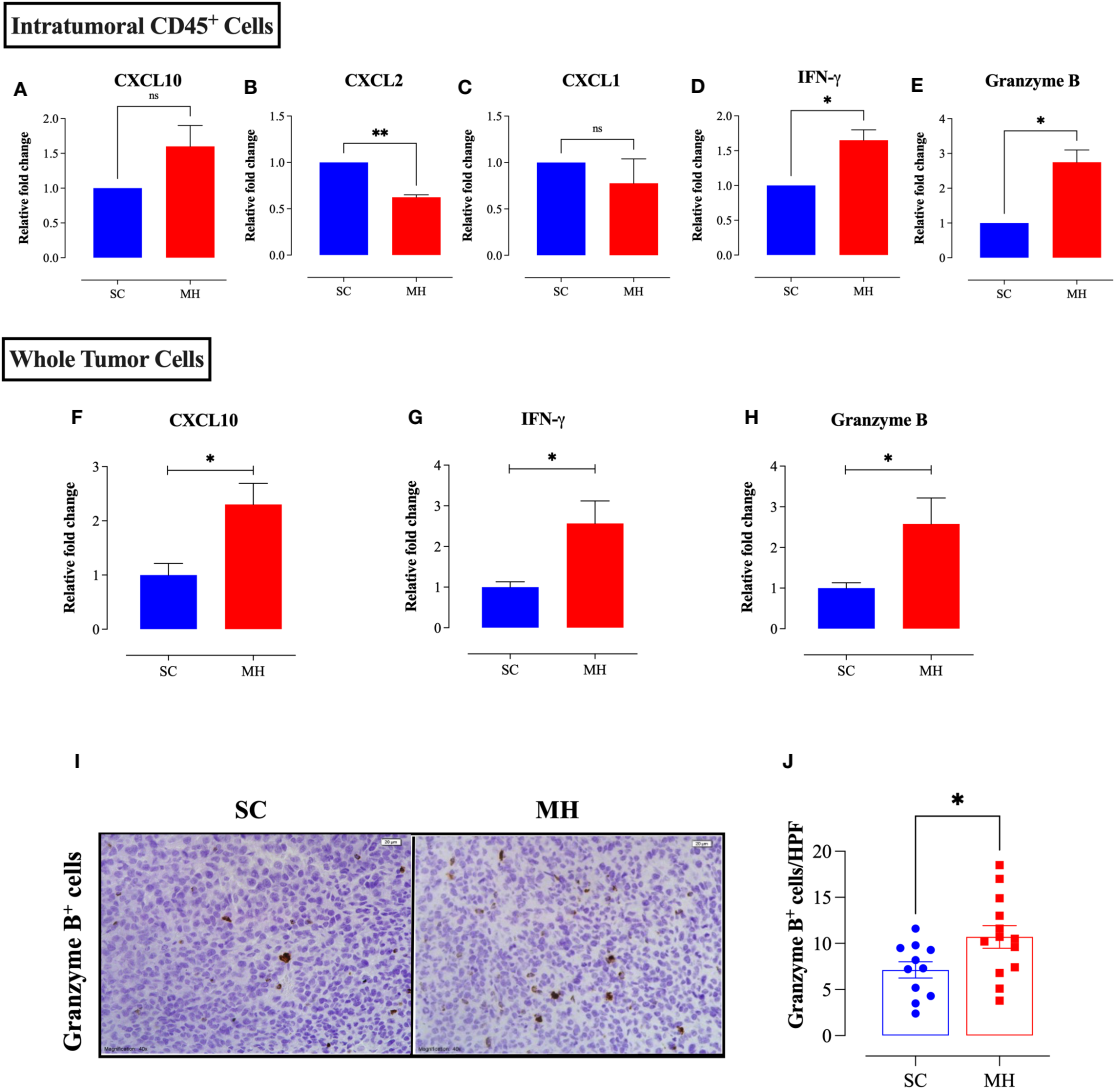
MH treatment alters the expression of chemokines and anti-tumor effector molecules within the tumor microenvironment. CT26 tumor tissues were excised from SC-treated and MH-treated mice on day 21 post-implantation. CD45^+^ cells were purified from a pool of tumor cells obtained from 4 tumors per group. RNA was extracted from the purified CD45^+^ cells and used to assess the mRNA levels of CXCL10 **(A)**, CXCL2 **(B)**, CXCL1 **(C)**, IFN-γ **(D)**, and granzyme B **(E)**. The data are expressed as means ± SEM of 2 replicates per group and are representative of 2 independent experiments. **(F–H)** RNA was extracted from whole tumor tissue and assessed for the relative expression of CXCL10 **(F)**, IFNγ **(G)** and granzyme B **(H)** genes. **(I, J)** Tissue sections were analyzed by immunohistochemistry to quantify the number of granzyme B^+^ cells. Representative images at 40× magnification (scale bar 20 μm) are presented for each group **(I)**. Quantitative estimation of the number of granzyme B^+^ cells/HPF (high-power field) is shown in panel **(J)**. The values for individual mice and mean ± SEM are shown (SC: n=11, MH: n=13), pooled from 3 independent experiments. Asterisks denote statistically significant differences between the MH-treated and SC-treated groups. *p* values were calculated using the unpaired Student’s t-test, * (*p* ≤ 0.05), ** (*p* ≤ 0.01), and ns (no statistical significance, *p* > 0.05).

To elucidate the mechanism by which MH modulates the cellular components of the TME and exerts the observed anti-tumor response, RNA was extracted from purified, intratumoral, CD45^+^ cells, or whole tumor tissue, of SC-treated or MH-treated mice. The RNA was then used to determine the gene expression levels of key inflammatory chemokines and cytotoxic effector molecules by qRT-PCR. At the level of tumor-infiltrating leukocytes, MH treatment led to a small (1.6-fold) but insignificant increase in the expression level of the chemokine CXCL10 (**Figure 5A**). At the whole tumor level, there was also a 2.3-fold increase in the expression of CXCL10 in MH-treated mice (**Figure 5F**; *p* = 0.0131). CXCL10 is secreted in response to IFN-γ and preferentially regulates the recruitment of inflammatory T lymphocytes (43). The expression levels of CXCL2 and CXCL1 chemokines, which are potent chemoattractants that control the recruitment of polymorphonuclear leukocytes in inflammation and tissue injury (44), were also examined. qRT-PCR results indicated a statistically significant 1.6-fold decline in the expression level of CXCL2 in MH-treated mice (**Figure 5B**). A trend toward a decrease in the expression levels of CXCL1 was also observed, but this did not reach statistical significance (**Figure 5C**). These findings may underlie the observed reduction in the proportion of intratumoral Ly6G^+^ granulocytes following MH treatment.

MH treatment also resulted in a significant increase in the expression levels of IFN-γ (~1.7-fold) and granzyme B (~2.8-fold), as detectable at the level of TILs (**Figures 5D, E**). Both of these mediators are secreted by effector immune cells to induce tumor cell apoptosis (45, 46). A significant increase (2.6-fold) in the expression of IFN-γ (*p* = 0.0148) and granzyme B (*p* = 0.0261) was also observed at the level of the whole tumor tissue (**Figures 5G, H**). Consistent with these findings, immunohistochemical staining of tumor tissues indicated an increase in the number of granzyme B-positive cells following MH treatment (**Figures 5I, J**), reflecting the presence of activated, anti-tumor, cytotoxic lymphocytes, most likely T cells and/or NK cells.

### Therapeutic efficacy of oral MH against implanted CRC tumors

**Figure 6:**
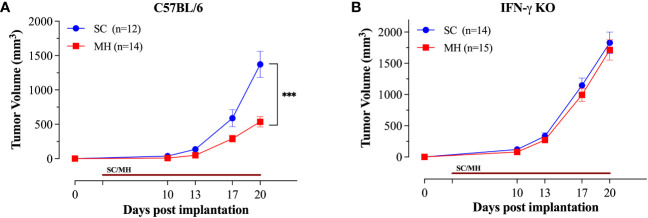
Therapeutic treatment with oral MH retards tumor growth in an IFNγ-dependent manner. Normal C57BL/6 **(A)** or IFNγ-deficient **(B)** mice were implanted with tumor cells and were orally gavaged daily starting on day 3 post implantation with 70% SC or 70% MH for 3 consecutive weeks. Each data point represents the mean ± SEM of the indicated mice within each experimental group, pooled from 2 independent experiments. Asterisks denote statistically significant differences between the SC-treated and MH-treated groups. *p* values were calculated using 2-way ANOVA, *** (*p* ≤ 0.001).

Having demonstrated the capacity of oral MH as a preventive treatment against cancer growth, we assessed its potency in a therapeutic model. Mice were implanted with MC38 CRC cells and oral MH was subsequently administered on a daily basis starting on day 3 post implantation. The data affirm that daily MH administration leads to a significant inhibition of tumor growth in normal, immunocompetent, mice (**Figure 6A**). The effect of MH on tumor growth was first apparent at about 10 days after the initiation of treatment. In sharp contrast, MH-induced curtailment of tumor growth was completely abrogated in IFNγ-deficient mice (**Figure 6B**), demonstrating mechanistically that MH most likely exerts its immunomodulatory effect via the activation of the IFNγ pathway.

### Oral MH induces changes in gut microbiota

**Figure 7:**
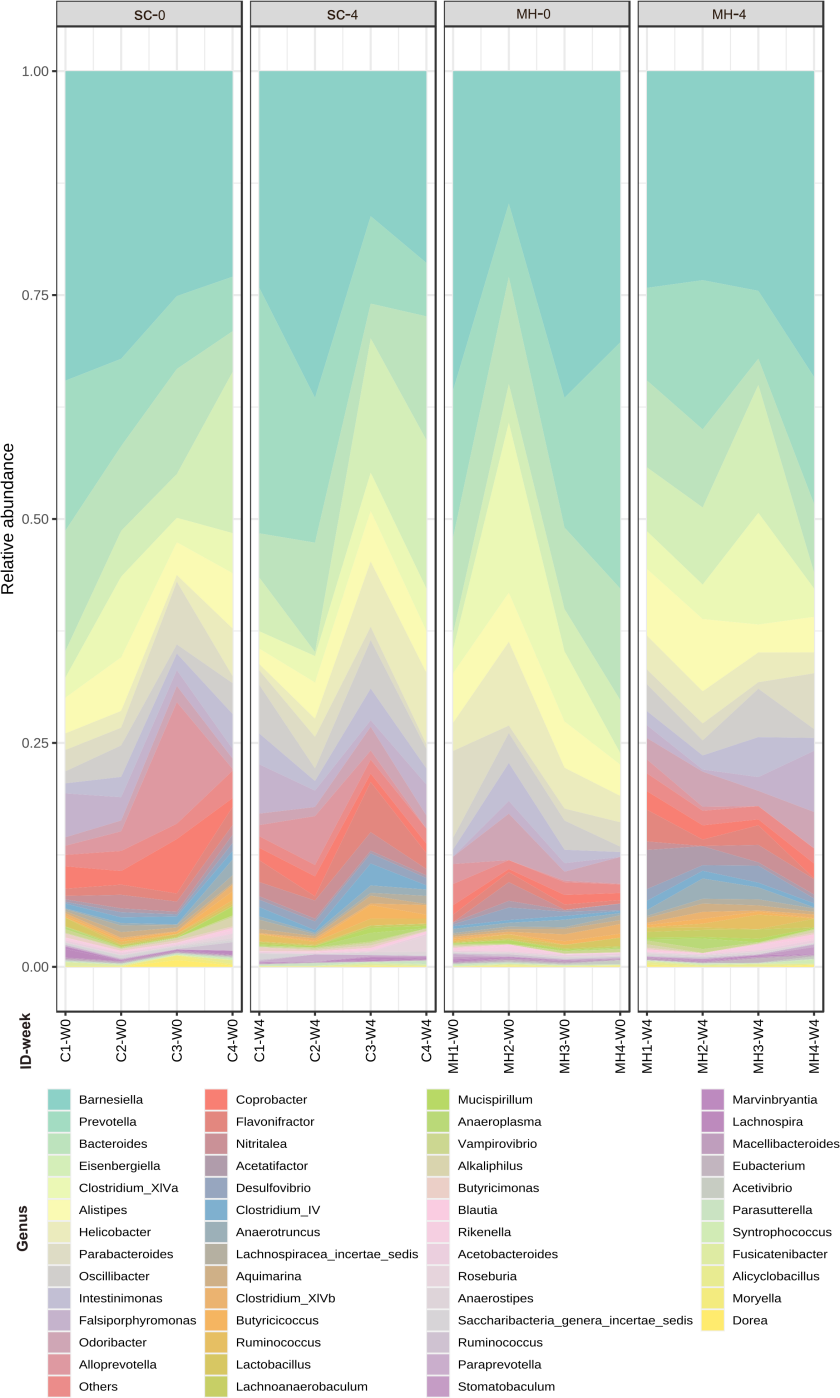
Relative abundance of genera detected at the baseline (week 0 before treatment) and in week 4 after treatment with SC or MH. Data shown represent the relative abundance of the genera listed, in each mouse investigated in this study before and after treatment.

**Figure 8:**
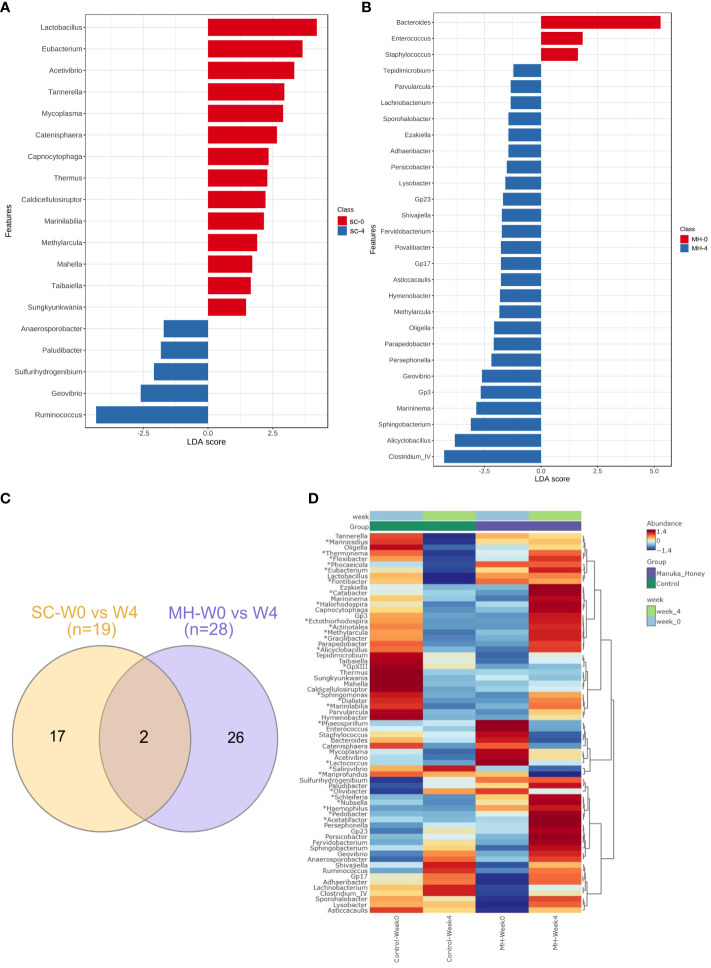
Microbiota variations after 4-week treatment. LDA analysis comparing genera pre and post-treatment with CS **(A)** and MH **(B)** showed depleted (red) and enriched (blue) in week 4 post-treatment. Venn diagram **(C)** of shared and unique genera that were significantly different pre- and post-treatment for each of the SC and MH groups. The heatmap **(D)** shows microbiota fingerprints in each group before and after treatment, with significantly altered genera. Asterisks (*) represent genera with significant differences between MH and CS after 4 weeks of treatment, while the rest are those with significant difference in week 0 compared to week 4 in either treatment group.

We hypothesized that oral administration of MH could induce changes in gut microbiota that would underlie the enhanced anti-tumor immune responses observed in these mice. To address this possibility, we determined the composition of gut microbiota in fecal samples collected from mice either before treatment or after 4 weeks of treatment with MH or SC solution. Microbiota were profiled at the genus level to detect the alterations in abundance and diversity caused by each treatment. The stacked area plot (**Figure 7**) shows the relative abundances of genera ranked based on their prevalence in the samples (listed below in the graph) collected from mice before and after treatment with SC or MH. Variations were obvious among the samples. The microbiota fingerprint in the control group was maintained between week 0 and week 4. As for the MH group, microbiota profiles looked more homogenous after treatment and with more similarity compared to the variability seen in week 0. To identify the genera with significant differences before and after each treatment, linear discriminant analysis (LDA) was done. As shown in **Figures 8A, B**, treatment with either SC or MH caused significant changes in microbiota profiles, with depletion of some genera (red color in the graphs) and enrichment of others (blue color in the graphs). These findings confirm that the microbiota were changed after 4 weeks of either treatment. It is noteworthy that treatment with SC caused depletion of *Lactobacillus* which is generally considered a beneficial bacteria. In sharp contrast, MH treatment caused depletion of pathogenic bacteria namely, *Staphylococcus, Enterococcus*, and *Bacteroides.*

Next, we investigated if microbiota alteration induced by treatment was similar in MH versus SC groups. Significantly changed microbiota identified by LDA analysis in MH and SC groups (from **Figures 8A, B**) were compared, and Venn diagram (**Figure 8C**) was used to identify unique and shared genera. Most of the genera altered in response to treatment with MH and SC were unique for each group. Only two genera were shared between the two groups, namely *Methylarcula* (more in MH in week 4, and more in SC in week 0, i.e. enriched after treatment with MH and depleted after treatment with SC), and *Geovibrio* (less in week 4 in both groups, i.e. reduced due to treatment with both SC and MH). Moreover, we compared the genera detected in MH and SC groups after 4 weeks of treatment to explore microbiota differences between these groups post-treatment. The heatmap also shows the genera with significant difference between week 0 and week 4 after each treatment (shown in **Figures 8A, B**) and reveals the distinct microbiota fingerprints per group. As shown in **Figure 8D**, significant differences were found in the genera after 4 weeks of treatment with SC or MH (marked with asterisk in the heatmap). For example, MH induced the depletion of several genera, including *Bacteroides*, *Staphylococcus* and *Enterococcus*, which have been described to have pro-tumorigenic activities (47–51). In contrast, two of the microbiota genera enriched after MH treatment were *Ruminococcus* and *Clostridium* cluster IV, both of which encompassing members that have been recognized for their anti-tumorigenic potential (52, 53). The LDA analysis of the significantly different genera is shown in (**Supplementary Figure 6**). The relative abundance of each significantly different genus among the groups is shown in (**Supplementary Figure 7**).

**Figure 9:**
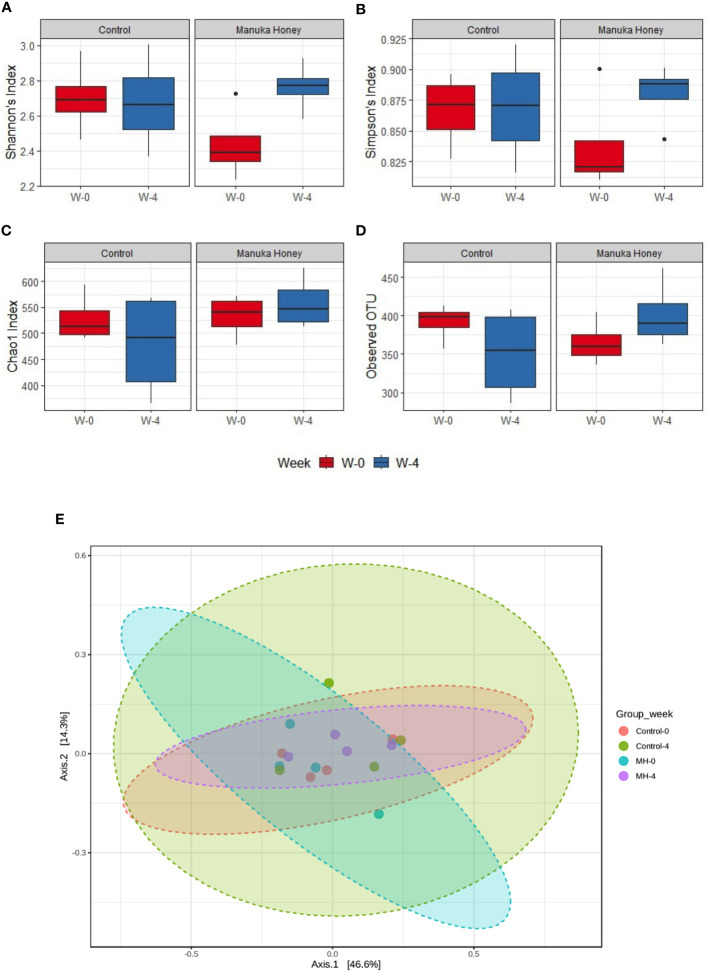
Microbiome diversity. **(A–D)** Show alpha diversity comparison between samples grouped based on treatment type and duration, using Shannon’s index **(A)**; Simpson’s index **(B)**; Chao1 index **(C)**, and observed species **(D)**. Box plots show Q1-median-Q3 with data range. Black dots are outlier values. Principal coordinates (PCo) analysis plots of beta diversity measured by Bray Curtis index **(E)** for control group and MH group in week 0 vs week 4. All groups are color-coded, and each dot represents an experimental mouse in each group. Although the difference was not statistically significant, (PERMANOVA: F-value: 0.92753; R-squared: 0.13389; p-value: 0.461), week 4 (post-treatment) was distinct from week 0 (pre-treatment) in the MH group.

Additionally, we have explored the effect of MH and SC treatment on microbiota diversity. The difference was not statistically significant in alpha and beta diversity (**Figures 9A–E**), but the only exception was seen in Shannon’s index, which is a widely used alpha diversity metric. The latter index is the negative sum of proportional microbiota abundance multiplied by the log of its proportional abundance (54). It is generally useful in predicting diversity by reflecting both richness (the number of microbiota) and evenness (the uniformity of distribution of microbiota) within a community (55). Shannon’s index was significantly higher after treatment with MH for 4 weeks compared to the baseline in week 0 (**Figure 9A**), suggesting an increase in the richness and evenness of microbiota after treatment with MH. This effect was not evident after treatment with SC. Nevertheless, pairwise comparison between SC and MH in week 0 and week 4 did not reveal any significant difference between these groups. Altogether, our findings demonstrate that MH treatment led to distinct changes in microbiota composition that are significantly different from the effect of SC, with identification of key microbiota that were increased or decreased following treatment.

## Discussion

Previous reports from our laboratory highlighted the role of MH as an anti-cancer and immunomodulatory agent (6–8, 10, 11, 15). In the current study, we present compelling evidence demonstrating the capacity of orally-administered MH to boost anti-tumor immune defense against an implanted colon adenocarcinoma tumor. To the best of our knowledge, this is the first report to demonstrate mechanistically how preventative, or therapeutic, administration of MH can lead to alterations in the cellular landscape within the TME that promote a more effective anti-tumor immunity.

The present study highlights several novel findings regarding the potential use of MH as a preventative and therapeutic agent against cancer. First, *in vivo* experiments using the oral administration route demonstrated immunological alterations consistent with the induction of IFN signaling pathway. Second, the significance of MH-induced immunological alterations was highlighted in two separate murine CRC models, where pre-treating mice with a daily oral dose of MH over 4 weeks resulted in a retardation of tumor growth. Third, MH-mediated tumor inhibition correlated with a series of cellular changes within the TME. Fourth, these intratumoral cellular alterations were accompanied by changes in the expression levels of various immunomodulatory chemokines and inflammatory cytokines. Fifth, MH-induced improvement in anti-tumor immune responses was also evident when used in a therapeutic regimen and was completely dependent on IFNγ. Lastly, MH treatment modulated gut microbiota composition, enriching for a unique pattern of several bacterial genera and inducing a depletion of pathogenic bacteria. Notwithstanding these findings, a major limitation of the present study is the use of ectopic tumor models. The utilization of a genetic, spontaneous, CRC model would increase the relevance of our findings. While the proportions of immune cells in the peripheral lymphoid tissues remained unaltered after MH treatment, there was a noticeable increase in their activation status, as evidenced by the upregulation of Sca-1 expression on lymphoid cells in the MLNs, ILNs, and spleens. Sca-1 is an interferon-inducible protein (36–38), that is upregulated as a result of inflammatory responses (56). Thus, the data indicate that oral MH treatment most likely triggers an inflammatory response that ultimately leads to an enrichment of type I/II interferons in both the gut and periphery. Induction of the IFN response may well be triggered when pathogen-associated molecular patterns (PAMPs) interact with membrane-bound pathogen recognition receptors (PRRs), such as Toll-like receptors (TLRs). TLRs recruit the MyD88 adaptor protein upon binding with their respective ligands, which leads to downstream activation of NF-kB, interferon regulatory factor 3 (IRF3), and interferon regulatory factor 7 (IRF7) transcription factors, which are responsible for inducing type I interferons (57, 58). Our previous findings showed that the immunostimulatory effect of MH following its intraperitoneal administration was significantly reduced in the absence of the MyD88 protein, indicating that TLRs may be involved in MH-triggered response (15). Since TLRs are involved in inducing type I IFN, it is plausible that oral administration of MH stimulates, directly or indirectly, type I IFN through a TLR-dependent pathway. However, further investigation is required to verify this hypothesis. Utilizing mice with known TLR defects would be useful in confirming this notion and elucidating the underlying mechanism in finer detail.

Alternatively, the enhancement in anti-tumor immune responses by MH could be related to the demonstrated changes in gut microbiota. Microbial dysbiosis is known to stimulate the host immune system, particularly T cell immune responses (59, 60). A previous study showed that an increase in Sca-1 expression on lymphoid cells in MLNs and peripheral tissues is linked to microbial dysbiosis in B cell-defective mice. The dysbiosis in the gut mucosal environment leads to type I IFN enrichment in CD8^+^ T cells, resulting in increased anti-tumor immunity (61). Honey has been shown to acquire protective prebiotic effects due to the presence of oligosaccharides and polyphenols as major constituents (62). MH was shown to improve the growth of probiotic bacteria while inhibiting the growth of pathogens (63). Animal studies have shown that oral administration of 2.2g/kg (44 mg/mouse) of MH to mice for 4 weeks leads to alterations in the concentrations of short-chain fatty acids (64). A small clinical study, involving 20 healthy individuals aged 42-64 years, was conducted to establish the safety of daily MH consumption, particularly in regard to allergic responses or changes in gut microbiota (65). The consumption of 20g of MH (UMF 20+) daily for 4 weeks did not result in any significant changes in gut microbiota (65). The authors postulated that the prebiotic effects of MH may have been masked by various factors such as the interaction with other dietary components, the storage conditions of honey, and the relatively low dosage of MH used. This suggests that factors beyond simply the actual dose may have influenced the impact of MH. In the current study, we demonstrate the capacity of orally-administered MH to induce significant changes in gut microbiota composition. It is worth noting that the dose used in our study is a comparatively higher dose than previously used (approximately 140 mg/mouse, equivalent to a human dose of 39.8 g). Our findings suggest that daily consumption of MH could boost immunity against development of cancer in at risk population. While this is perhaps a rather simplistic view, given the complexity of the process of cancer development and progression and the multitude of different cancer types, it is nevertheless an important message from the point of view of MH potentially being an important immunomodulatory agent.

We investigated the impact of oral MH treatment on implanted tumors. By focusing on using a preventative treatment regimen, we could delineate the immunomodulatory effect of MH from its anti-tumor effect. Our findings demonstrated a 44% reduction in tumor growth compared to the control group. To gain insights into the underlying mechanism of this effect, we analyzed the immune system components of CT26 tumors by flow cytometry. Our analysis revealed that the observed inhibition of tumor growth was associated with a significant enhancement in CD45^+^ hematopoietic cell infiltration into the tumor tissue. Further investigation revealed that treatment with MH increased the proportion of intratumoral cytotoxic and helper T cells. T cells have been shown to play a crucial role in inducing anti-tumor immune responses (66). Cytotoxic effector CD8^+^ T cells can directly recognize and kill cancer cells by releasing cytotoxic molecules, such as granzymes and perforin, as well as pro-inflammatory cytokines like IFN-γ and TNF-α (67). Similar to CD8^+^ T cells, CD4^+^ T cells secrete pro-inflammatory cytokines with direct anti-tumor effects (68). Additionally, CD4^+^ T cells play a crucial role in activating and expanding CD8^+^ T cells through the secretion of IL-2, which promotes their proliferation and activation. Moreover, CD4^+^ T cells license dendritic cells (DCs) to activate CD8^+^ cells by either cross-presenting tumor antigens to CD8^+^ T cells or inducing the production of cytokines and costimulatory molecules (69–71). Our findings revealed that MH treatment not only increased the infiltration of intratumoral T cells but also enhanced their cytotoxic potential, as shown by the elevated levels of IFN-γ and granzyme-B in purified CD45^+^ immune cells from tumor tissue.

In addition to alterations in TILs, we have observed changes in the intratumoral myeloid populations in response to MH treatment. Specifically, there was a reduction in the proportion of CD11b^+^ myeloid cells, accompanied by a significant decrease in the proportion of Ly6G^+^ granulocytes. In addition, MH treatment may enhance the antigen-presenting capacity of intratumoral myeloid cells, as suggested by their increased expression of MHC class II proteins. It is important to acknowledge the limitations inherent in our analysis of myeloid cells in the TME. These cells represent a very heterogeneous and complex subpopulations with different functions (72). The use of only CD11c marker to identify dendritic cells is limited given the fact that these are quite heterogeneous in nature. Furthermore, another limitation in our analysis of the intratumoral myeloid cells is the absence of additional cell markers to distinguish M1 and M2 macrophages. Myeloid cells, including TAMs, DCs, tumor-associated neutrophils (TANs), and myeloid-derived suppressor cells (MDSCs), are the most abundant immune cells in the TME, and their heterogeneity allows them to exert both pro-tumor and anti-tumor effects during tumor development and progression (73). The role of MDSCs in suppressing anti-tumor immunity and supporting the proliferation of tumors has been well-documented (39). In our study, MH-mediated tumor inhibition was associated with a significant reduction in the percentages of intratumoral Ly6G^+^ myeloid cells, which resemble granulocytic-MDSCs that are known to contribute to tumor growth promotion and immune response suppression. Various studies have reported the presence of granulocytic-MDSCs within the tumors and organs of CT26-bearing mice (39, 74, 75). CT26 tumors produce proinflammatory mediators and factors that contribute to the development and expansion of granulocytic-MDSCs in both primary tumors and distant organs (76, 77). This alteration in myeloid populations could explain the tumor-inhibiting effects of MH treatment. An increase in both the proportion and functional ability of cytotoxic T cells following MH treatment suggests that the suppressive effect of granulocytic-MDSCs on T cells is reduced. However, to verify this, it is crucial to directly evaluate the immunosuppressive capacity of intratumoral Ly6G^+^ CD11b^+^ cells by performing cellular function assays. Our results also indicated an increase in the expression of MHC-II proteins on the intratumoral myeloid cells, implying that type I and/or type II interferons could be responsible for this induction (34, 35). These findings indicate that these cells are potentially more able to act as antigen-presenting cells to CD4^+^ helper T cells, hence augmenting anti-tumor T cell responses (66).

One major mechanism through which tumors avoid the immune response is by downregulating MHC class I, thereby decreasing their recognition and elimination by cytotoxic CD8^+^ T cells (78). A promising approach to enhancing the efficacy of anti-tumor therapies involves restoring the expression of MHC class I through type I/II IFN stimulation (79). In the current study, MH treatment enhanced the expression of MHC-I on the CD45^-^ tumor cells, indicating the involvement of type I and/or type II interferons in this induction. The increase in MHC-I expression is consistent with the observed increase in TILs and IFNγ expression in MH-treated mice. The increased CXCL10 expression, which is also triggered by IFN-γ, may regulate the recruitment of inflammatory T lymphocytes (43). Our findings also demonstrated that MH treatment reduces CXCL2 expression, which plays a crucial role in recruiting intratumoral granulocytic MDSCs and promoting their pro-tumor immunosuppressive function (44, 80). These findings suggest that oral MH treatment enhances the immunogenicity of CT26 tumor cells, making them more susceptible to cytotoxic T cell-mediated killing.

In line with our findings, previous studies demonstrated the potential of natural products like polyphenols to restructure the immunosuppressive microenvironment of tumors and hinder tumor growth (81, 82). These natural products have been shown to downregulate the percentages of immunosuppressive cells, such as MDSCs, Tregs, and M2-MACs, while promoting the proportions and function of anti-tumor effector T cells like CD8^+^ T cells, CD4^+^ T cells, and NK cells (81–83). Given that MH comprises a variety of polyphenols, it is perhaps not unreasonable to suggest that these bioactive substances contribute to the elicitation of the observed anti-tumor immune responses following MH treatment. Alternatively, oral administration of MH could alter anti-tumor immunity through changing gut microbiota-derived metabolites. Nutritional regulation of these metabolites and their influence on the immune system has been recently reviewed (84). Although much remains to be elucidated, there is evidence for individual metabolites acting to either improve responses to cancer therapy, such as indole-3-acetic acid (85), or mitigate against high-fat-diet-mediated progression of intestinal tumors, such as butyrate (86). Moreover, butyrate was shown to inhibit gastric tumors by reducing the expression of immunosuppressive factors, such as PD-L1 and IL-10 (87). In the context of colorectal cancer, a recent study demonstrated that oral administration of *Lactobacillus plantarum* CBT could effectively inhibit the growth of colorectal cancer in noth orthotopic as well as ectopic preclinical mouse models (88). Given the evidence that MH could effect changes in gut microbiota content, it would be extremely beneficial to characterize the relative changes in the metabolite abundance with a view of uncovering their influence on cancer growth and response to therapy.

Current studies have shown that certain members of the intestinal microbiota can facilitate colorectal carcinogenesis by generating carcinogenic microbial metabolites and secreting oncogenic virulence factors (89). It is worth noting that some of the bacterial genera that were significantly different after 4-week treatment with MH were previously reported to have an impact on tumorigenesis. For the genus *Enterococcus*, which was depleted after MH treatment, previous studies have reported an association between some species of *Enterococcus* (e.g. *E. faecalis*) and gastrointestinal tumorigenesis related to its interaction with immune cells. Studies in IL-10 deficient mice found that these bacteria can cause macrophage polarization to M1 phenotype, resulting in inflammation and DNA damage of intestinal epithelial cells (90, 91). Furthermore, *Enterococcus* can secret tumor-stimulating metabolites with proliferative and angiogenic effects on CRC (51). As for *Bacteroides*, which was also depleted after MH treatment, some species such as *B. fragilis* can contribute to oncogenic transformation in the colon by producing enterotoxins which can induce c-Myc expression and cellular proliferation in intestinal epithelial cells (92). Recent experimental evidence confirmed that *Bacteroides*-driven colitis can promote colon tumorigenesis. Colonization with enterotoxigenic *B. fragilis* can induce mucosal IL-17 production with subsequent events leading to tumor formation, a process that was ameliorated by IL-17 neutralization (48). Specifically, *Bacteroides* toxin (fragilysin) triggers an IL-17 immune response that activates NF-κB signaling in colonic epithelial cells, leading to pro-tumoral myeloid cells infiltration in the colon (93). Finally, for the genus *Staphylococcus*, which was also depleted after MH treatment, surveillance studies in patients with CRC revealed that some species, such as *S. lugdunensis*, were associated with colon carcinoma (49). Moreover, bidirectional functional effects of *Staphylococcus* species on carcinogenesis have been proposed (50), mostly driven by the many immunoregulatory factors produced by *Staphylococcus* member species. Interestingly, some staphylococcal nucleases have been recognized as oncoproteins (94).

As for the microbiota enriched after MH treatment, many of them are associated with health-promoting effects, and some are recognized as anti-tumorigenic bacteria. For instance, *Ruminococcus* was enriched after MH treatment. Bacteria from this genus are secondary bile acid producers (95). These metabolites are able to suppress colon carcinogenesis through modulation of signaling pathways in colon cancer cells (52, 96). Moreover, bile acids produced by these bacteria have strong antimicrobial properties and can modulate the gut microbiome by selectively eliminating pathogens, supporting the growth of other health-promoting bacteria (97). *Clostridium* cluster IV which encompasses several butyrate producers, was significantly enriched after MH treatment. *Clostridium* cluster IV includes four members, namely *C. leptum, C. sporosphaeroides, C. cellulosi and Faecalibacterium prausnitzii* (98). All these bacteria have potent probiotic characteristics which are essential for intestinal homeostasis, thus providing protection against cancers. For instance, *F. prausnitzii* has proven anti-tumorigenic and anti-proliferative effect by inhibiting the formation of abnormal colonic crypt foci in animal models of CRC. Furthermore, the application of *F. prausnitzii* reduced the level of lipid peroxidation in colonic tissues, which is also protective against CRC (53).

There is mounting evidence indicating that the gut microbiota play a crucial role in cancer development and response to anti-cancer therapies (99, 100). Analysis of the gut microbiota of CRC patients has shown that certain bacteria, such as *Streptococcus gallolyticus*, *Fusobacterium nucleatum*, *Escherichia coli*, *Bacteroides fragilis*, and *Enterococcus faecalis*, are more prevalent in CRC patients compared to the normal population, while the levels of other genera like *Roseburia*, *Clostridium*, *Faecalibacterium* and *Bifidobacterium* decrease in CRC patients (101). Experimental evidence from preclinical as well as clinical studies demonstrated that gut microbiota plays a critical role in influencing the response to anti-cancer therapies. For instance, in a murine melanoma model, the presence of commensal *Bifidobacterium* was linked to differences in response to immune checkpoint inhibitors (ICI), and fecal microbiota transplantation improved the anti-tumor effectiveness of PD-L1 blockade (102). Clinical studies have further revealed that the composition and diversity of the gut microbiota can predict a favorable response to ICI immunotherapy, with specific bacterial strains such as *Ruminococcus*, *Akkermansia muciniphila*, and *Bifidobacterium* being present in the gut microbiome of ICI-responsive patients (103, 104). Therefore, manipulating the gut microbiome may have broad potential in cancer prevention and treatment.

**Figure 10:**
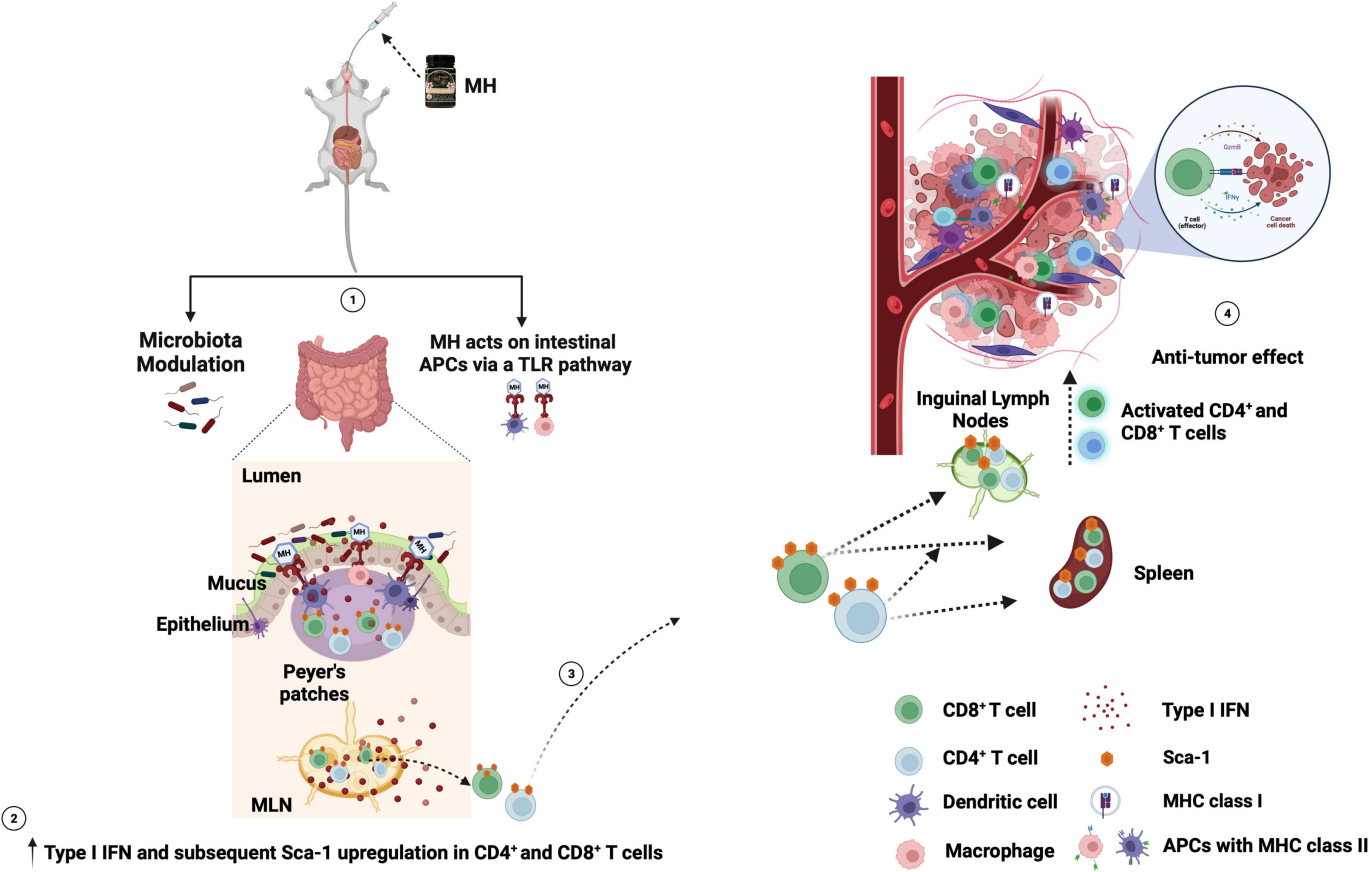
Schematic diagram of the proposed mechanism of oral MH on anti-tumor immune response. (1) MH treatment either modulates the gut microbiota or induces the TLR pathway. This leads to (2) the enrichment of type I IFN and the subsequent upregulation of Sca-1 on the CD4^+^ and CD8^+^ T cells in the gut environment. (3) The Sca-1^+^ CD4^+^ and CD8^+^ T cells migrate from the gut to the periphery (spleen and ILNs). (4) these preactivated T cells then migrate to the TME and induce anti-tumor immune responses. Figure adapted from (61) and created with BioRender.com.

Overall, our study indicates that oral administration of MH has the potential to activate the immune system and enhance anti-cancer immune responses in a preclinical model of CRC. Although the full mechanistic details remain unknown, our findings suggest that pretreatment with MH can activate lymphoid cells in mucosal and peripheral tissues, thereby facilitating a preactivated ready-to-respond state, that is most likely contributing to the superior anti-tumor immunity. Most importantly, MH treatment appears to promote anti-tumor immunity even when given therapeutically post tumor implantation. MH appears to enrich for type I/II IFN signature by either altering the gut microbiota, or via a hitherto unknown mechanism, leading to the upregulation of Sca-1 on CD4^+^ and CD8^+^ cells in the gut, and their subsequent migration from the gut to the periphery. These T cells possess superior effector potential, ultimately promoting anti-tumor immune responses. The increased efficacy is linked to a series of immunological alterations within the TME, resulting in the suppression of tumor growth. The proposed mechanism of action for oral MH treatment is summarized in **Figure 10**. Validation of the proposed mechanism is required. For example, the use of mice pre-treated with antibiotics to deplete their microbiota would provide crucial evidence for a role for the microbiota in the observed MH-induced enhancement in anti-tumor immune responses. Furthermore, the potential of combining MH treatment with another modality, such as chemotherapy or immunotherapy, would be immensely rewarding for the ultimate goal of improving the efficacy of anti-cancer therapy.

## Data availability statement

The data supporting the findings of this study are available within the article and its supplementary material. The 16S rRNA sequencing data are deposited in the Sequence Read Archive (SRA) in the National Center for Biotechnology Information (NCBI) database under Accession No. PRJNA1063249. Further inquiries can be directed to the corresponding author.

## Ethics statement

The animal study was approved by Institutional Animal Research Ethics Committee of the United Arab Emirates University. The study was conducted in accordance with the local legislation and institutional requirements.

## Author contributions

BA: Conceptualization, Formal analysis, Funding acquisition, Project administration, Supervision, Validation, Visualization, Writing – original draft, Writing – review & editing. RM: Formal analysis, Investigation, Methodology, Visualization, Writing – original draft. II: Formal analysis, Investigation, Methodology, Writing – review & editing. YM: Formal analysis, Investigation, Methodology, Writing – review & editing. AsA: Formal analysis, Investigation, Methodology, Writing – review & editing. GB: Formal analysis, Investigation, Methodology, Writing – review & editing. FA: Formal analysis, Investigation, Methodology, Visualization, Writing – original draft, Writing – review & editing. AbA: Formal analysis, Investigation, Writing – review & editing. MF: Formal analysis, Supervision, Validation, Visualization, Writing – review & editing.
