# Supplementary material for: Oral administration of Manuka honey induces IFNγ-dependent resistance to tumor growth that correlates with beneficial modulation of gut microbiota composition
Source: Front Immunol. 2024 Feb 20;15:1354297. doi: 10.3389/fimmu.2024.1354297 (PMC10912506; doi:10.3389/fimmu.2024.1354297)
Supplement: Supplementary file 1 [file DataSheet_1.pdf]

Supplementary Material

Oral administration of Manuka honey induces IFNγ-dependent resistance to tumor growth that correlates with beneficial modulation of gut microbiota composition

Razan J. Masad[1], Ienas Idriss[1], Yassir A. Mohamed[1], Ashraf Al-Sbiei[2], Ghada Bashir[1], Farah Al-Marzooq[1], Abeer Al-Tahrawi[3], Maria J. Fernandez-Cabezudo[2,4] & Basel K. al-Ramadi[1,4,5*]

[1] Department of Medical Microbiology and Immunology, College of Medicine and Health Sciences, United Arab Emirates University, Al Ain, United Arab Emirates.

[2] Department of Biochemistry and Molecular Biology, College of Medicine and Health Sciences, United Arab Emirates University, Al Ain, United Arab Emirates.

[3] Department of Pathology, College of Medicine and Health Sciences, United Arab Emirates University, Al Ain, United Arab Emirates.

[4] Zayed Center for Health Sciences, United Arab Emirates University, Al Ain, United Arab Emirates.

[5] ASPIRE Precision Medicine Research Institute Abu Dhabi, United Arab Emirates University, Al Ain, United Arab Emirates.

[*] Corresponding Author

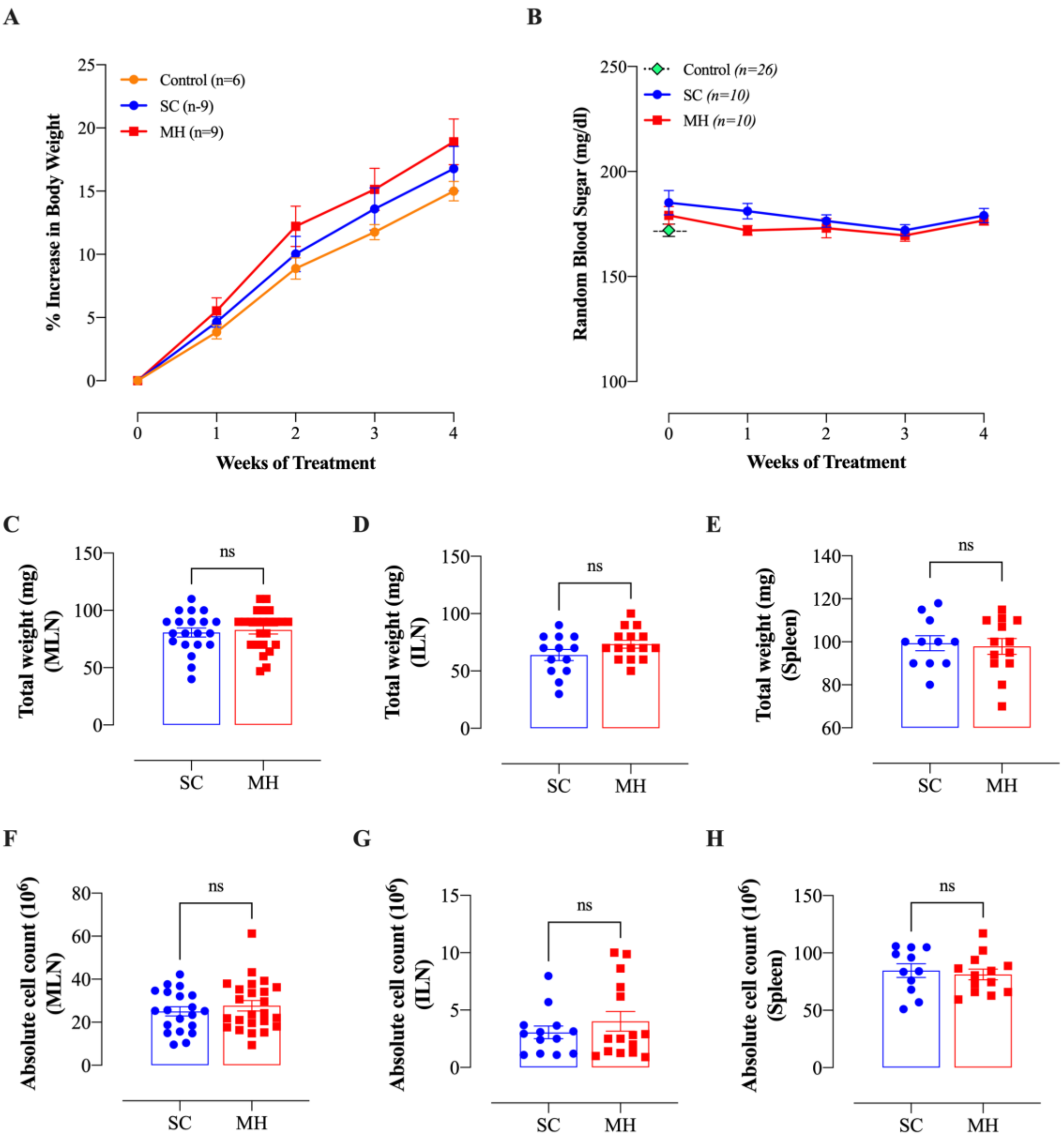

Supplementary Figure 1. MH treatment does not alter the body weight, blood glucose levels, and the total weights and absolute cell counts of different peripheral lymphoid tissues. BALB/c mice were orally gavaged with filtered water, 70% SC or 70% MH for 4 consecutive weeks. Mice were then euthanized, and their MLNs, ILNs, and spleens were collected for further investigation. (**A**) Percent increase in body weight in mice treated with water, SC, or MH. Data is representative of 3 independent experiments. (**B**) Blood glucose levels of mice treated with SC or MH. Data is pooled from 2 experiments. (**C-H**) Total weights (**C-E**) and absolute cell count (10^6) (**F-H**) of MLNs, ILNs, and spleens, respectively, following the treatment period. The values for individual mice and mean ± SEM are shown. Data in **(C, F)** is pooled from 5 independent experiments. Data in **(D, G)** is pooled from 4 independent experiments. Data in (**E, H**) is pooled from 3 independent experiments. p values were calculated using the unpaired Student's t-test, ns (no statistical significance, $p > 0.05$).

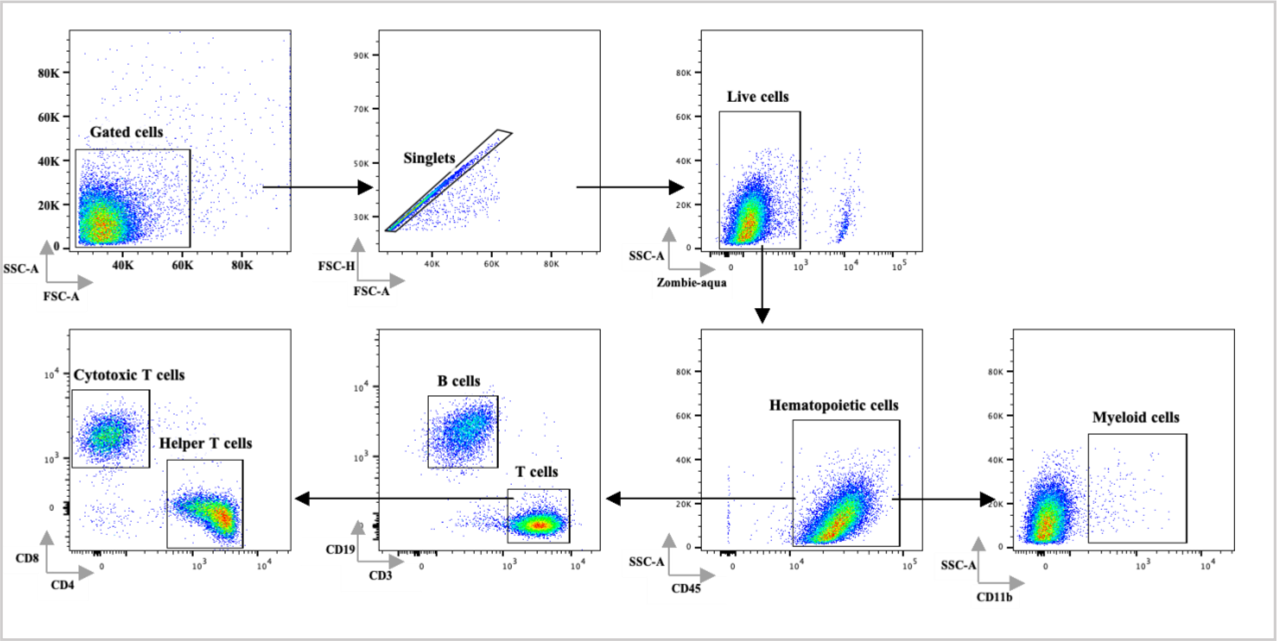

Supplementary Figure 2. The gating strategy used to identify the cell populations in peripheral lymphoid organs. Single-cell suspensions of MLNs, ILNS, or spleens were stained with different fluorophore-conjugated antibodies and analyzed by flow cytometry. Following the exclusion of doublets and non-viable cells, immune cells were identified as positive for the CD45 marker. Among the CD45$^+$ cells, the myeloid cells (CD11b$^+$), B cells (CD19$^+$), and T cells (CD3$^+$) were identified. The T cells subpopulations were identified as cytotoxic T cells (CD8$^+$) and helper T cells (CD4$^+$).

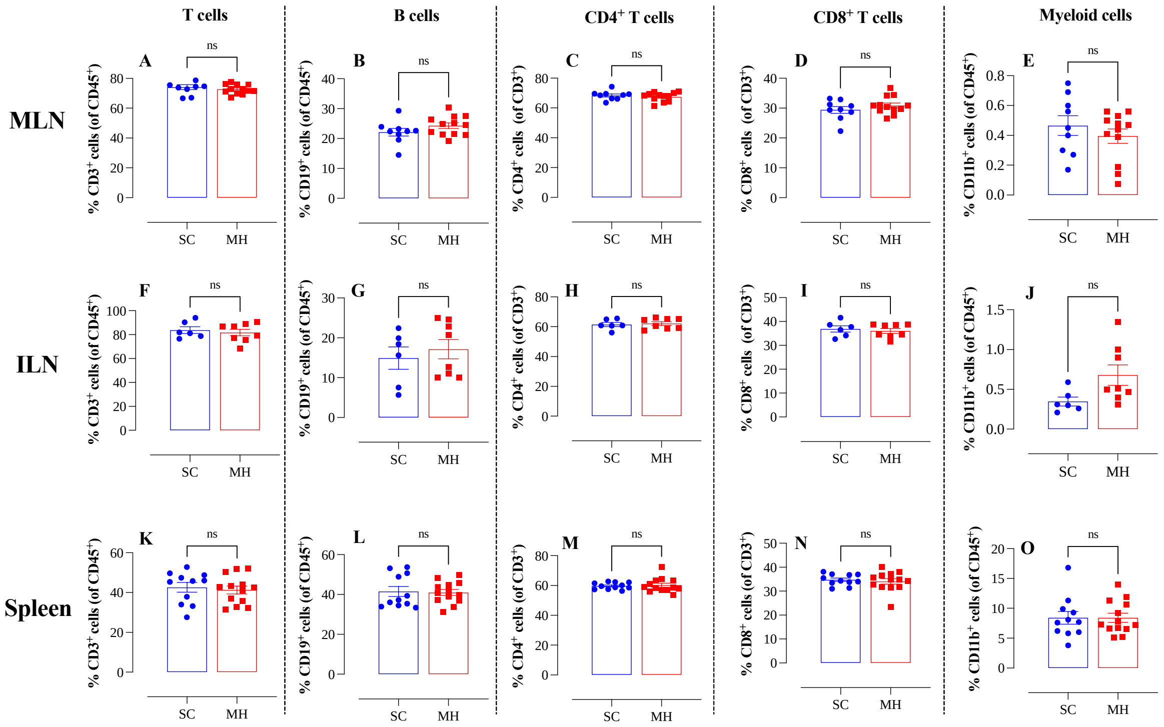

Supplementary Figure 3. Cellular distribution in peripheral lymphoid tissues following MH treatment. BALB/c mice were orally gavaged with either SC or MH for 4 weeks. Following treatment, lymphoid organs were processed for flow cytometry analysis. Cells from MLNs (panels **A-E**), ILNs (panels **F-J**), and spleens (panels **K-O**) were analyzed for the presence of $CD3^+$ T cells (**A, F, K**), $CD19^+$ B cells (**B, G, L**), $CD4^+$ helper T cells (**C, H, M**), $CD8^+$ cytotoxic T cells (**D, I, N**), and $CD11b^+$ myeloid cells (**E, J, O**). The values for individual mice and mean ± SEM are shown. The data shown is pooled from either 3 independent experiments (**A-E)** and (**K-O**) or 2 experiments (**F-J**). p values were calculated using the unpaired Student's t-test, ns (no statistical significance, $p > 0.05$).

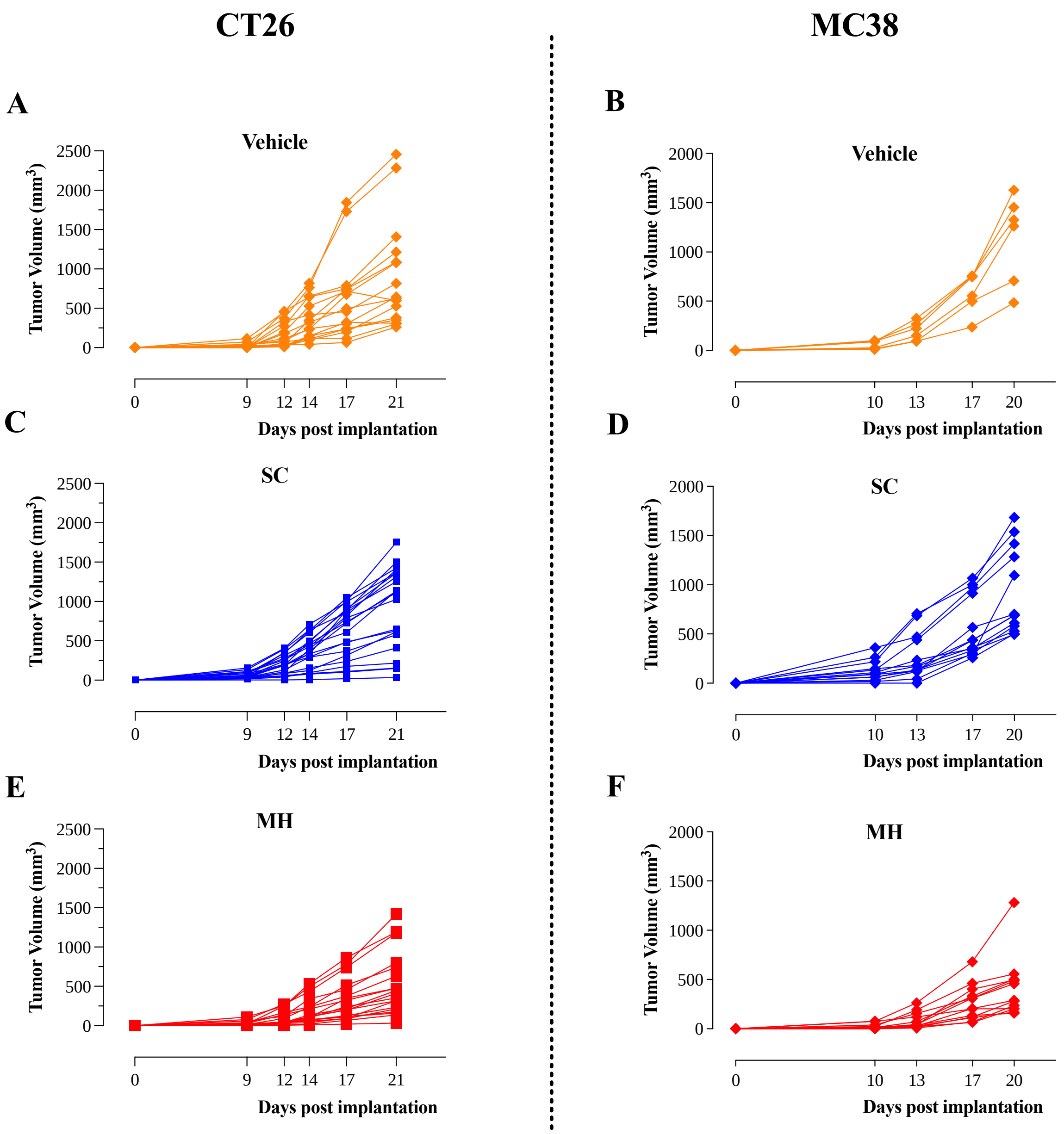

Supplementary Figure 4. Pre-treatment with oral MH retards the growth of CT26 and MC38 tumors. Mice were orally gavaged daily with filtered water (Vehicle; **A-B**), 70% SC (**C-D**) or 70% MH (**E-F**) for 4 consecutive weeks. Following the treatment period, CT26 or MC38 CRC cells were implanted and tumor growth was followed for the subsequent 3 weeks. Tumor growth in individual mice of the vehicle-, SC-, or MH-treated mice is shown for CT26 (**A**,**C**,**E**) and MC38 tumor model (**B**,**D**,**F**).

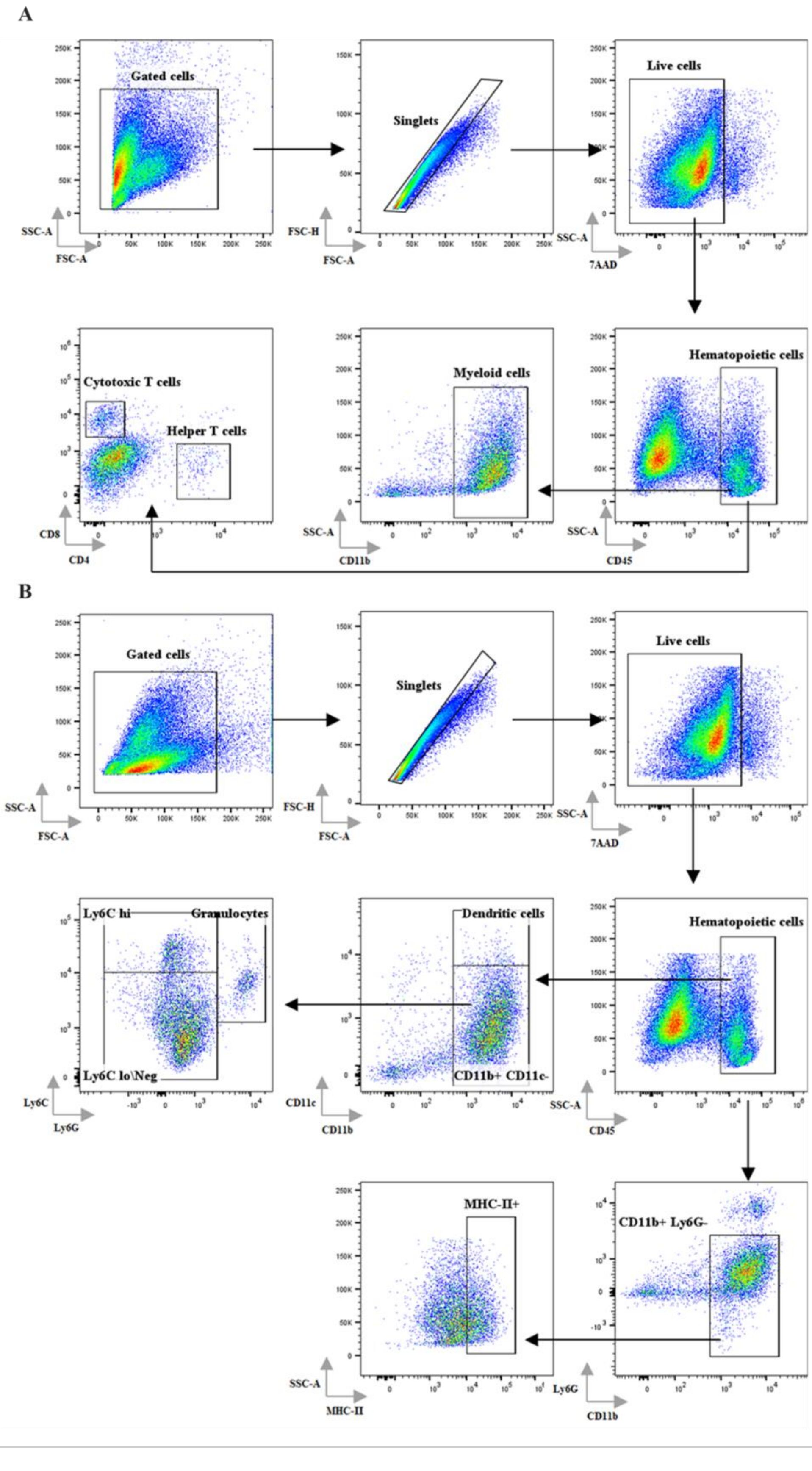
A
Gated cells
SSC-A
FSC-A
Singlets
FSC-H
FSC-A
Live cells
SSC-A
7AAD
Cytotoxic T cells
Helper T cells
CD8
CD4
Myeloid cells
SSC-A
CD11b
Hematopoietic cells
SSC-A
CD45
B
Gated cells
SSC-A
FSC-A
Singlets
FSC-H
FSC-A
Live cells
SSC-A
7AAD
Ly6C hi
Granulocytes
Ly6C lo\Neg
Ly6C
Ly6G
Dendritic cells
CD11b+ CD11c-
CD11c
CD11b
Hematopoietic cells
SSC-A
CD45
MHC-II+
SSC-A
MHC-II
CD11b+ Ly6G-
Ly6G
CD11b

Supplementary Figure 5. The gating strategies used to identify the intratumoral cellular populations. Single-cell suspensions were stained with different fluorophore-conjugated antibodies and analyzed by flow cytometry. (A) Phenotype panel: Following the exclusion of doublets and non-viable cells, immune cells were identified as positive for the CD45 marker. Among the $CD45^{+}$ cells, the myeloid cells ($CD11b^{+}$), cytotoxic T cells ($CD8^{+}$), and helper T cells ($CD4^{+}$) were identified. Among the myeloid cells, the granulocytes ($Ly6G^{+}$) were identified. (B) Myeloid panel; Following the exclusion of doublets and non-viable cells, immune cells were identified as positive for the CD45 marker. Among the $CD45^{+}$ cells, the dendritic cells were identified as $CD11b^{+}$ $CD11c^{+}$. The rest of the myeloid cells ($CD11b^{+}$ $CD11c^{-}$) were identified as granulocytes ($Ly6G^{+}$), $Ly6C^{hi}$ cells, and $Ly6C^{lo/Neg}$ cells. Among the $CD45^{+}$ cells, the antigen-presenting cells ($CD11b^{+}$ $Ly6G^{-}$) were identified and examined for their MHC class II expression.

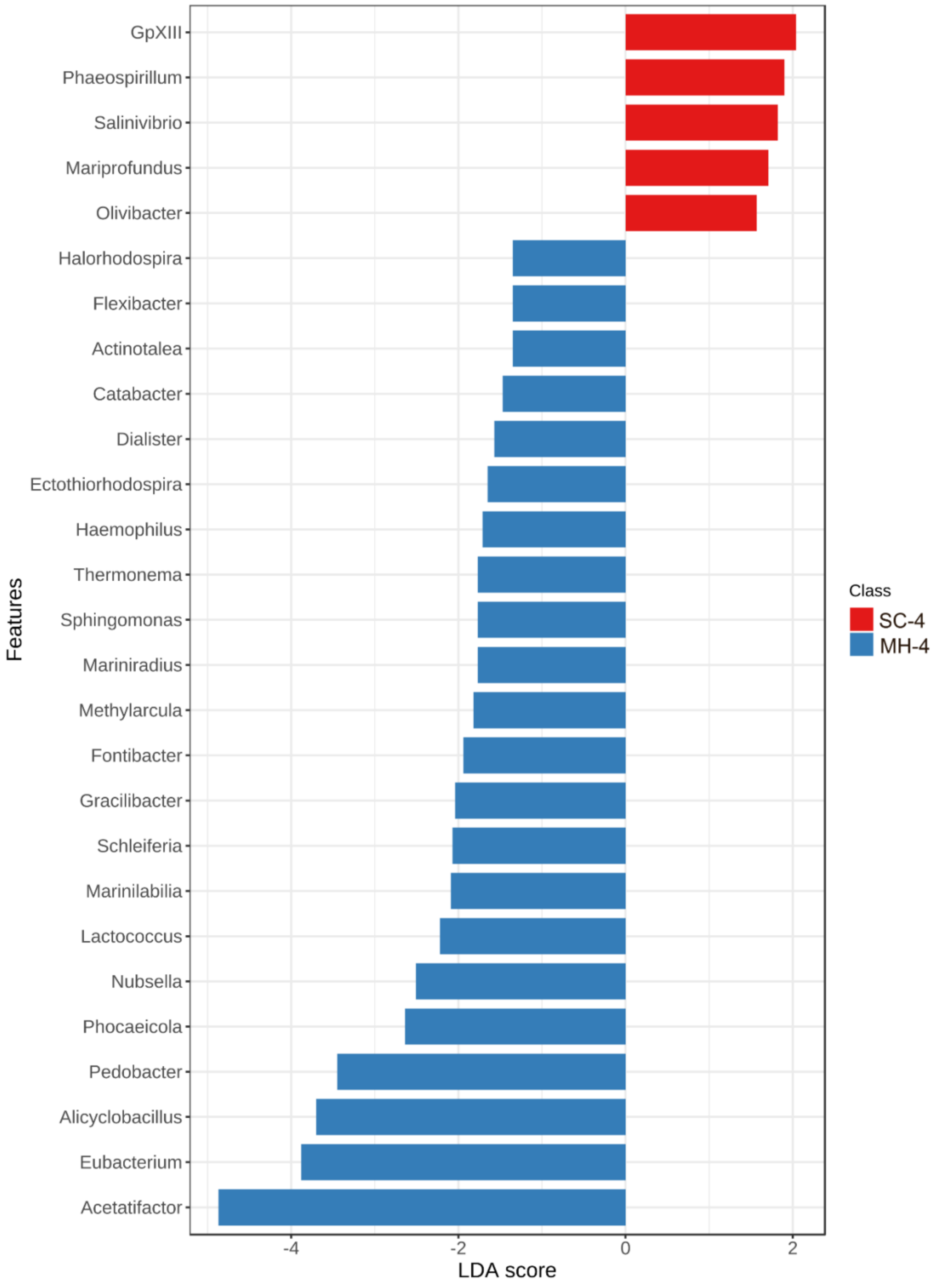

Supplementary Figure 6. LDA of significantly different genera of bacteria in MH vs SC groups after 4 weeks of each treatment. Treatment with MH caused the enrichment of 22 genera which were significantly more abundant than the control group. On the other hand, only 5 genera were enriched in the control group and depleted in MH group.

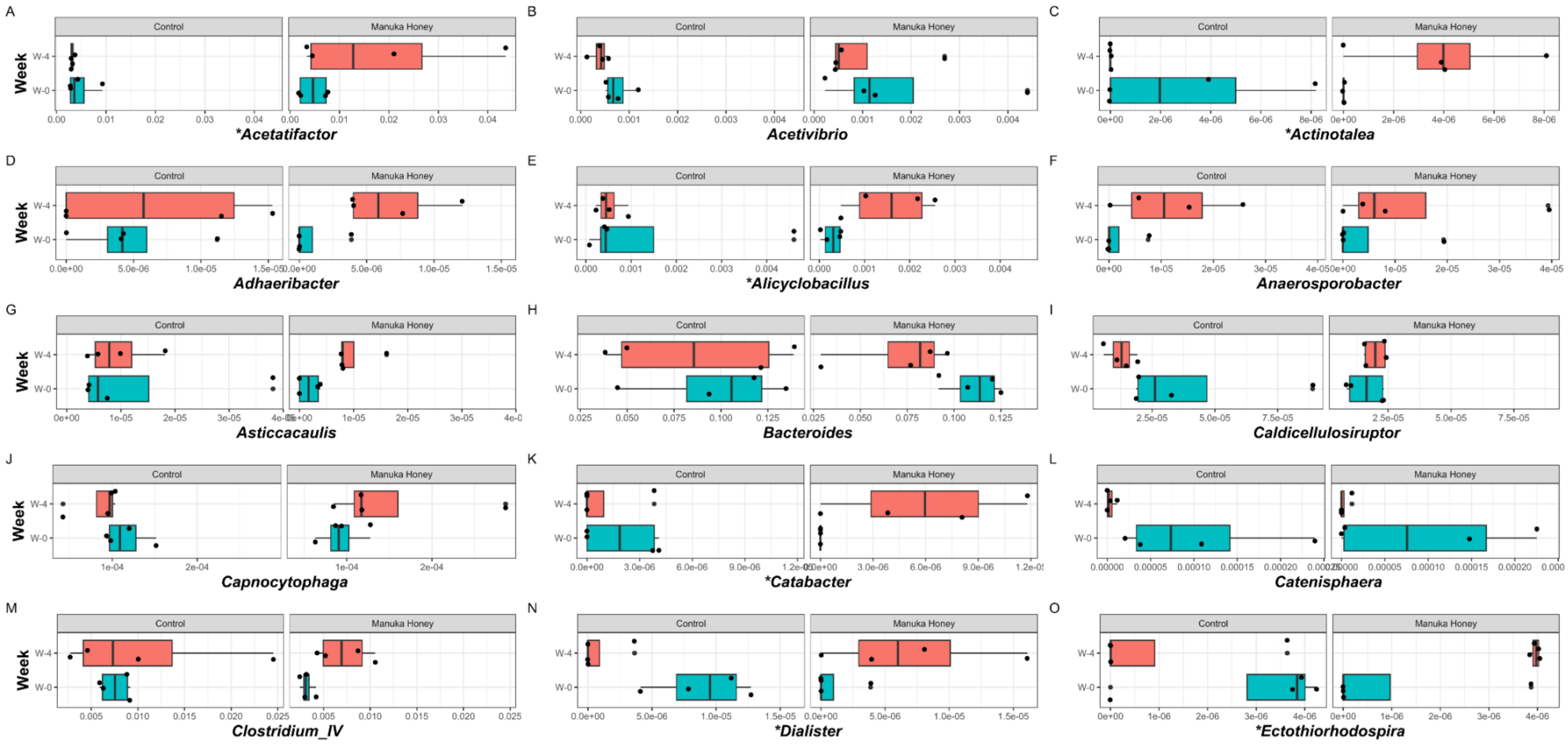
A
*Acetatifactor
B
Acetivibrio
C
*Actinotalea
D
Adhaeribacter
E
*Alicyclobacillus
F
Anaerosporobacter
G
Asticcacaulis
H
Bacteroides
I
Caldicellulosiruptor
J
Capnocytophaga
K
*Catabacter
L
Catenisphaera
M
Clostridium_IV
N
*Dialister
O
*Ectothiorhodospira
Control
Manuka Honey
Week
W-4
W-0

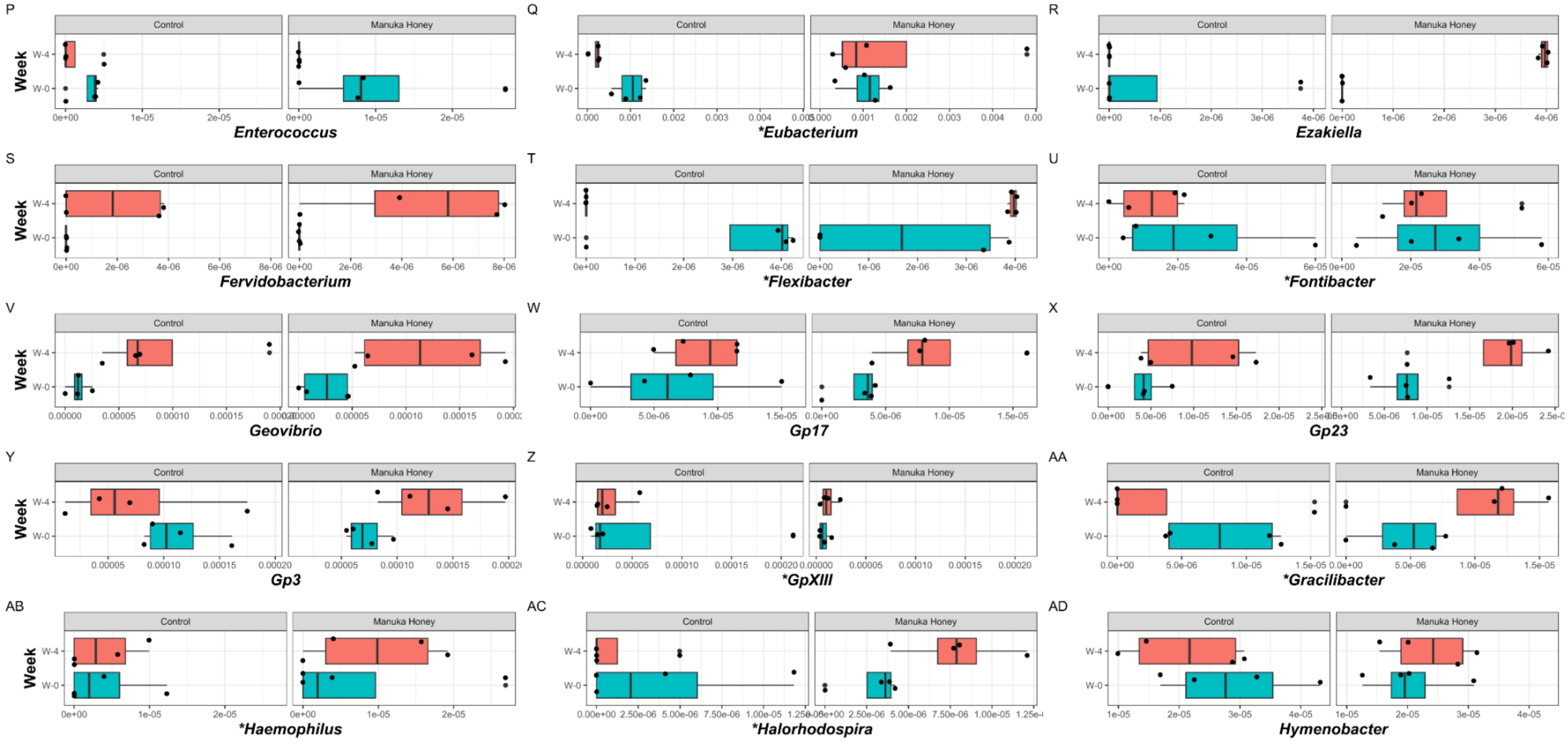
P
Enterococcus
Q
*Eubacterium
R
Ezakiella
S
Fervidobacterium
T
*Flexibacter
U
*Fontibacter
V
Geovibrio
W
Gp17
X
Gp23
Y
Gp3
Z
*GpXIII
AA
*Gracilibacter
AB
*Haemophilus
AC
*Halorhodospira
AD
Hymenobacter
Control
Manuka Honey
Week
W-4
W-0

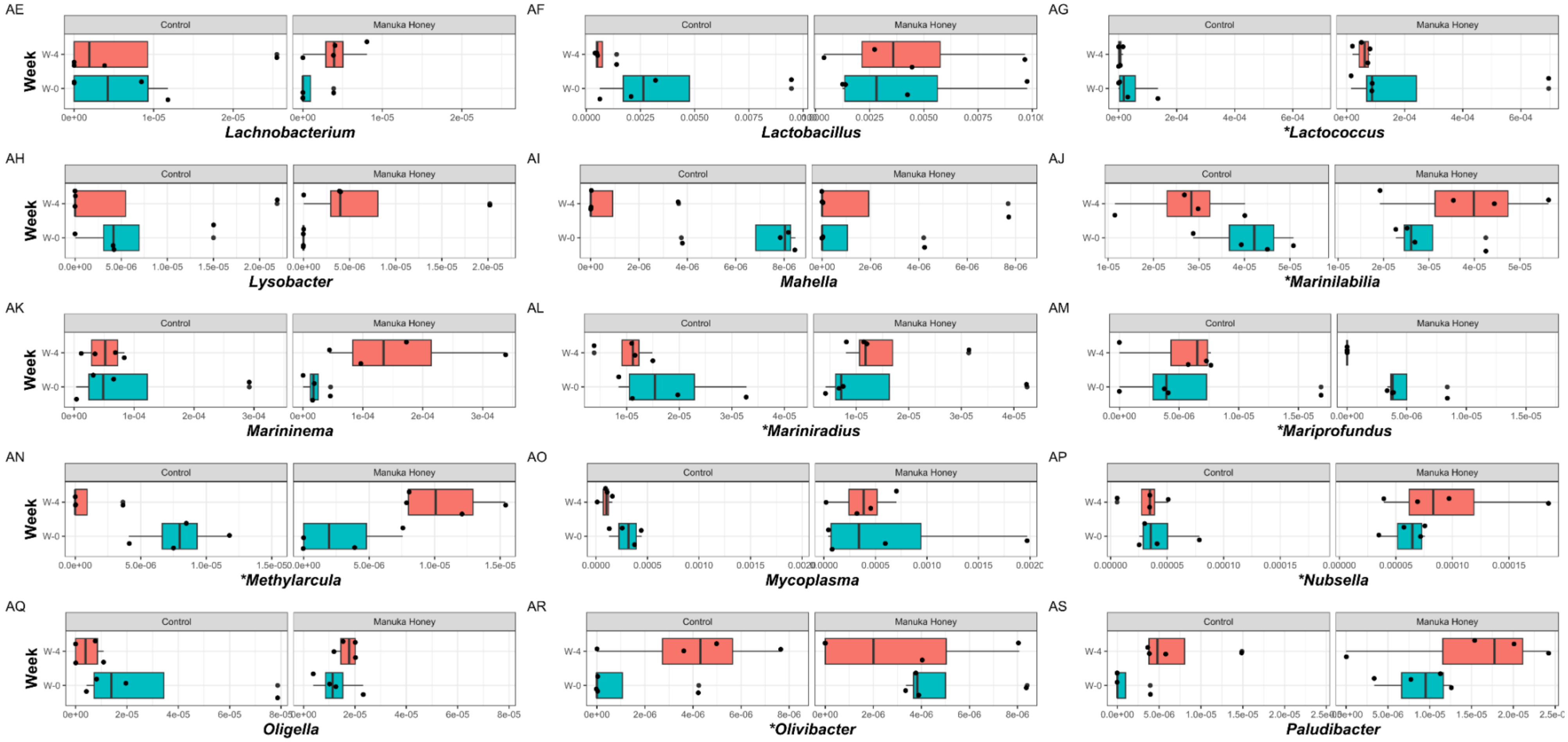
AE
Control
Manuka Honey
Week
W-4
W-0
Lachnobacterium
AF
Lactobacillus
AG
*Lactococcus
AH
Lysobacter
AI
Mahella
AJ
*Marinilabilia
AK
Marininema
AL
*Mariniradius
AM
*Mariprofundus
AN
*Methylarcula
AO
Mycoplasma
AP
*Nubsella
AQ
Oligella
AR
*Olivibacter
AS
Paludibacter

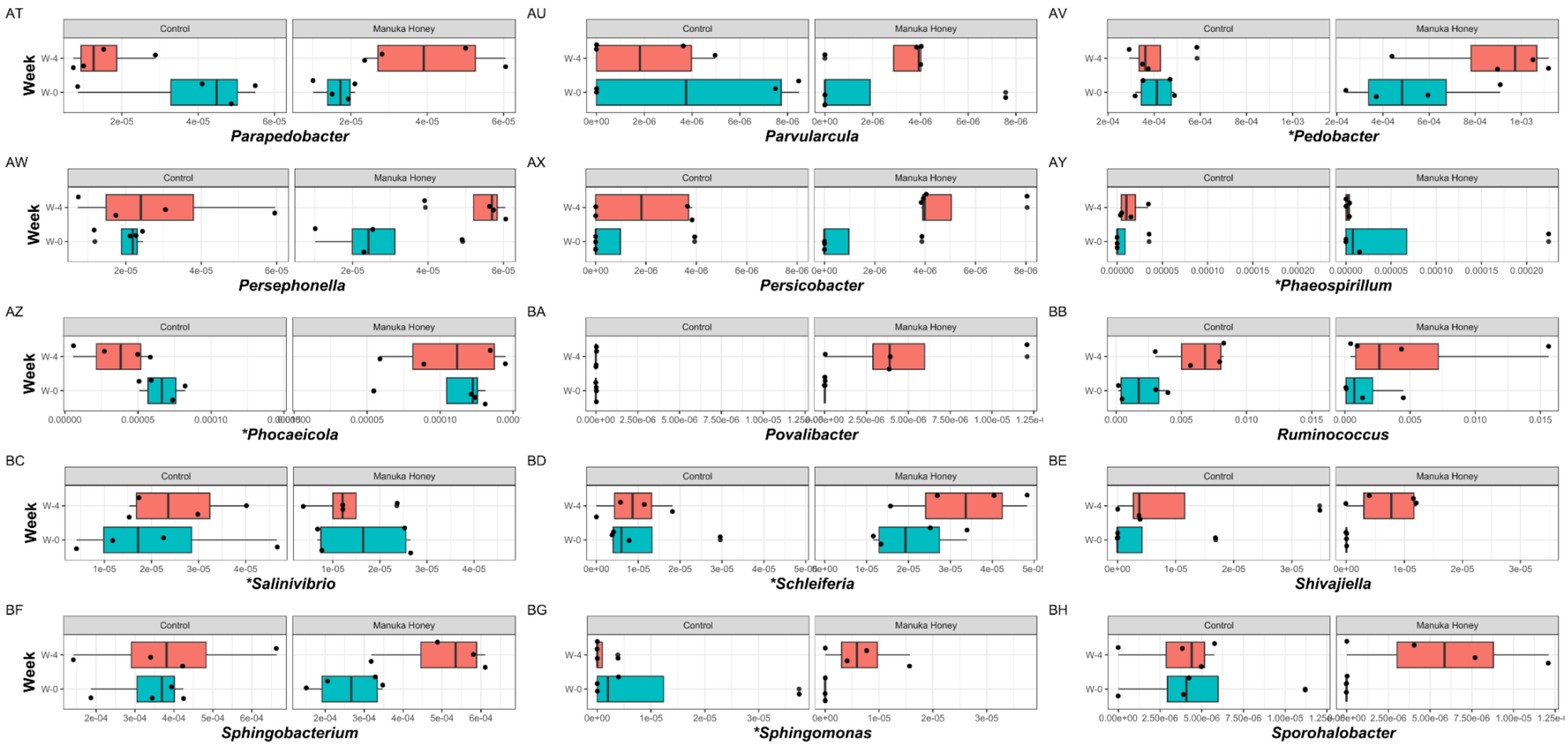
AT
Control
Manuka Honey
Week
W-4
W-0
Parapedobacter
AU
Parvularcula
AV
*Pedobacter
AW
Persephonella
AX
Persicobacter
AY
*Phaeospirillum
AZ
*Phocaeicola
BA
Povalibacter
BB
Ruminococcus
BC
*Salinivibrio
BD
*Schleiferia
BE
Shivajiella
BF
Sphingobacterium
BG
*Sphingomonas
BH
Sporohalobacter

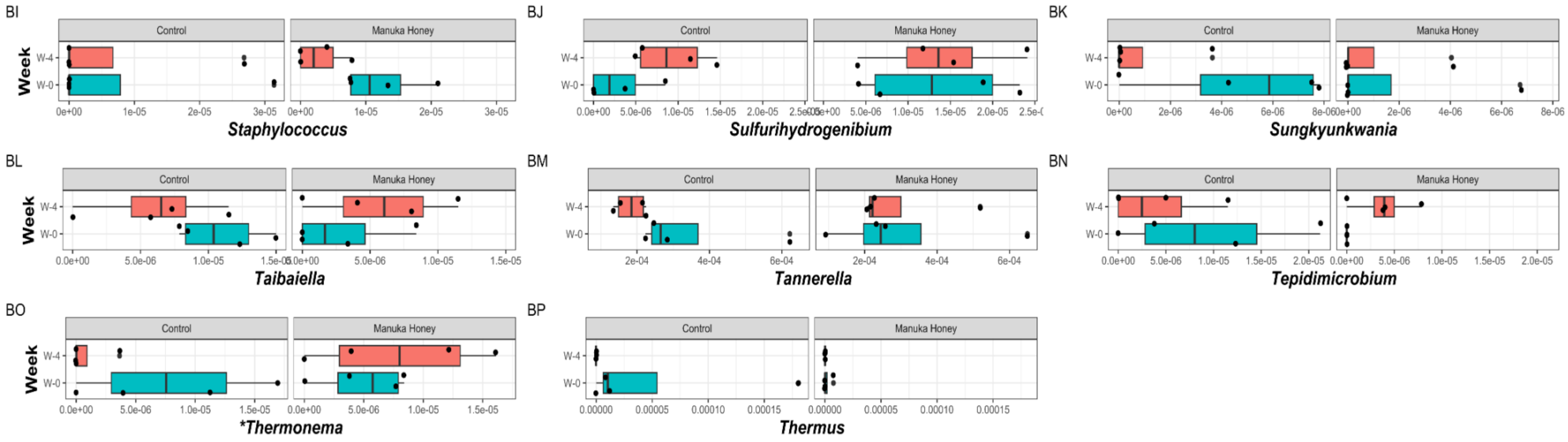

Supplementary Figure 7. The relative abundance of genera detected at significantly different level pre-treatment (week 0) and post-treatment (week 4) with Manuka honey and sugar control. Data from each mouse is shown in the figures as black dots. Genera marked with asterisk (*) are those detected at significantly different levels between Manuka honey and the control group after 4 weeks of treatment, while the rest are those with significant difference in week 0 compared to week 4 in either treatment group. The same genera are also shown in Figure 8 (heatmap) and supplementary figure 6 with LDA scores.
